# Chemical identification of an aggregation pheromone in the termite *Reticulitermes speratus*

**DOI:** 10.1038/s41598-020-64388-4

**Published:** 2020-05-04

**Authors:** Yuki Mitaka, Shigeru Matsuyama, Nobuaki Mizumoto, Kenji Matsuura, Toshiharu Akino

**Affiliations:** 1 Applied Entomology Laboratory, Department of Bioresource Field Sciences, Kyoto Institute of Technology, Kyoto, 616-8354 Japan; 2 Graduate School of Life and Environmental Sciences, University of Tsukuba, Ibaraki, 305-8572 Japan; 3 School of Life Sciences, Arizona State University, Arizona, AZ 85287-9425 USA; 4 Laboratory of Insect Ecology, Graduate School of Agriculture, Kyoto University, Kyoto, 606-8502 Japan

**Keywords:** Animal behaviour, Entomology, Behavioural ecology

## Abstract

Social behaviours in termites are regulated by sophisticated chemical communication systems. The majority of subterranean termites continuously forage for new wood resources to expand their nesting areas; an aggregation pheromone is presumed to regulate this process. However, the chemical components of this pheromone have never been determined. We identified the chemical properties of the aggregation pheromone that signals nestmate presence and induces arrest in the termite *Reticulitermes speratus*. The results of gas chromatography-mass spectrometry analyses and bioassays indicated that *R. speratus* worker release the pheromone to their nesting site. The pheromone consists of an aromatic compound (2-phenylundecane), cuticular hydrocarbons (pentacosane and heptacosane), fatty acids (palmitic acid and *trans*-vaccenic acid), and cholesterol; the pheromone induces long-term aggregation at new nesting and feeding sites. Although 2-phenylundecane alone attracted workers, the combination of all six compounds showed greater arrestant activity than 2-phenylundecane alone. This suggests that 2-phenylundecane functions as an attractant, whereas the remaining five components function as arrestants. Our results indicate that foraging worker termites produce a multi-component aggregation pheromone by combining a volatile hydrocarbon and non-volatile lipids with cuticular hydrocarbons. This pheromone enables rapid, long-lasting aggregation of termite workers, which contributes to efficient feeding and colonisation of new wood. Our work furthers the understanding of chemical communication systems underlying social assembly in social insects.

## Introduction

Aggregation is one of the most remarkable behaviours in the animal kingdom. Classically, aggregation behaviour has been viewed as evolutionarily advantageous, whereby individuals gain the benefits of protection, mate choice, and resource exploitation^1^. In gregarious insects, aggregation pheromones facilitate mate choice, group foraging, and collective gathering^2^. Aggregation pheromones gather conspecifics near the pheromone source, either by attracting them from a distance (attractant activity) or inducing passing conspecifics to remain at the pheromone source (arrestant activity)^3^. These pheromones can comprise a sole compound or multiple compounds. In sole-compound pheromones, a single compound plays the role of both attractant and arrestant;^4^ in multiple-compound pheromones, some compounds act as attractants and others act as arrestants^5^. Aggregation pheromones have been identified in various gregarious insects including cockroaches, stink bugs, bed bugs, locusts, fruit flies, bark beetles, longhorn beetles, and bees^2,6^. Social insects can also demonstrate self-assemblage; however, few studies have explored the proximate mechanisms of this aggregation behaviour^7^.

Termites are eusocial insects that occupy dead wood (their food source and habitat) at high densities. Termite colonies with a large number of siblings develop a remarkable caste-based division of labour; the worker caste collectively displays coordinated behaviours such as migration, nest construction, foraging, and recruitment^8^. Termites are typically classified into three categories based on their nesting habits: one-piece nesters (species which nest in and feed on a single piece of wood), multiple-piece nesters (species which nest in multiple wood pieces connected by underground tunnels and aboveground shelter tubes), and separate-piece nesters (species whose nests are physically separated from their food resources)^9,10^. In one-piece and multiple-piece nesters, the royal chamber typically acts as a central locus for foraging and feeding behaviours; the colony expands the nest area by feeding and foraging outward from the chamber, which is typically located deep in an inhabited wood piece. Therefore, workers and soldiers are densely distributed in the central and foraging areas^11^. When foraging workers discover a new food source, they gather their nestmates to that area; they first exploit the new foraging area, and later colonise it. Termites aggregate to a high density during this process because they require allogrooming and trophallaxis to exchange nutrients, gut symbionts, and immune substances with each other, which enables them to survive in oligotrophic and microbe-rich environments^8^. Thus, workers are susceptible to the Allee effect;^12^ rapidly formed yet long-lasting aggregation in newly colonised wood is essential to the ecological success of one-piece and multiple-piece nesting termites.

*Reticulitermes* termites are multiple-piece nesters with a sophisticated pheromone communication system^13–18^. Previous studies have suggested the existence of a chemical signal associated with worker aggregation, but this compound was not previously identified. Labial gland extracts derived from workers of *Reticulitermes santonensis* and *Schedorhinotermes lamanianus* have been shown to elicit gnawing and feeding behaviours, which result in the aggregation of feeding workers^19^. Other studies have shown that extracts of worker *Reticulitermes* termites have attractant activities to other workers^20^; the volatile components, 3-octanone and 3-octanol, play a role in corpse recognition cues and can induce aggregation-like conditions for corpse retrieval and cannibalism^21^. Moreover, termites have been shown to secrete a trail pheromone to lead and recruit passing nestmates to novel food locations, although this pheromone has no arrestant activity^22^. These studies indicate that chemicals trigger temporal aggregation. However, the main chemical components of the aggregation pheromone in *Reticulitermes*, which induces assembly on the pheromone source over an extended duration, remains unknown.

We compared the attraction activity of hexane extract fractions from worker *Reticulitermes speratus* and identified the pheromone components in active fractions. We then explored the optimal component ratio, dose dependency, and the persistence of aggregation activity of this pheromone.

## Results

### Attractive effects of fractions on workers

We identified pheromone components using a two-choice bioassay with multiple steps and gas-chromatographic analyses. In the two-choice bioassay, two pieces of filter paper were placed on the bottom of a plastic dish: a sample paper and a solvent control. In each bioassay, we compared the aggregation ratios (i.e., ratio of total number of workers on sample paper to total number of workers on solvent papers) in each treatment and also compared the proportions of workers on sample papers among treatments after 5 min, with longer times used in the later tests of dose-dependency.

**Figure 1:**
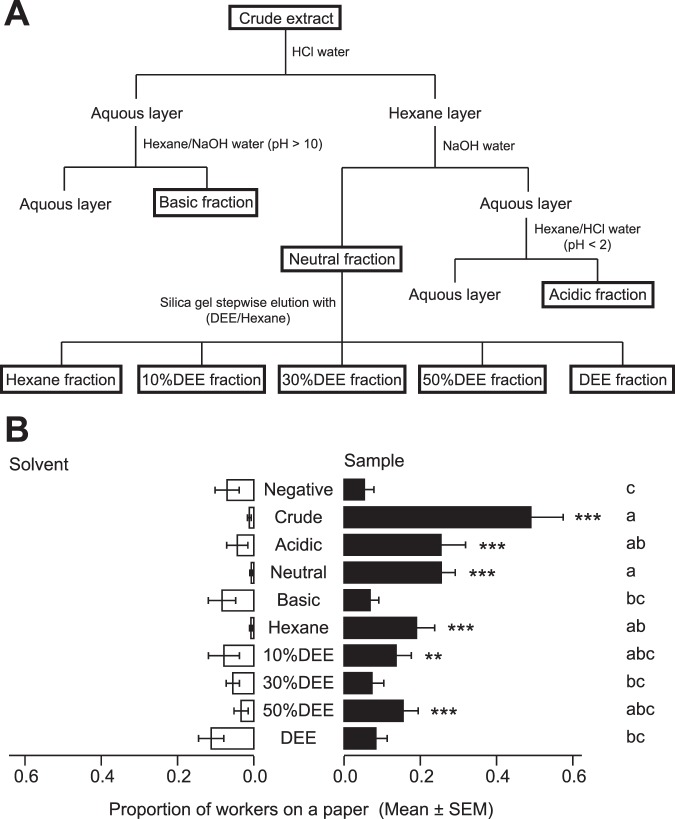
Attractant activities of crude worker extract and its fractions. (**A**) Separation scheme of the aggregation pheromone components extracted from worker termites through liquid−liquid extraction, followed by silica gel column chromatography (DEE: diethyl ether). (**B**) Bioassay results of the crude extract and its fractions after 5 min. Black bars indicate mean proportions of workers on sample papers and white bars indicate mean proportions of workers on solvent paper. Each treatment had 20 replicates (10 replicates × 2 colonies). Asterisks indicate significant differences between total numbers of workers on sample papers and total numbers of workers on solvent papers (binomial test with Bonferroni correction, *P < 0.05, **P < 0.01, ***P < 0.001). Different alphabets indicate significant differences in the mean of proportion of workers on sample papers among treatments (GLMM followed by Tukey HSD test, *P* < 0.05).

Initially, crude worker extract was fractionated into neutral, acidic, and basic fractions; the neutral fraction was further chromatographed into five consecutive fractions, eluted with *n*-hexane, three mixtures of diethyl ether (DEE) in *n-*hexane (10%, 30%, 50%), and DEE, respectively (Fig. 1A). When comparing total number of workers on the sample papers with total number of workers on the solvent papers in each treatment, the acidic, neutral, hexane, 10% DEE, and 50% DEE fractions attracted significantly more workers, relative to solvent papers, within 5 min (binomial test with Bonferroni correction, *P* < 0.01, Fig. 1B). Comparing the proportion of workers on sample papers among treatments, there were no significant difference among these fractions and the crude extract (a generalised linear mixed model [GLMM] followed by Tukey HSD test, *P* < 0.05). The main active components of the aggregation pheromone were therefore separated into the acidic, hexane, 10% DEE, and 50% DEE fractions.

### Chemical profile of fractions of worker extracts

**Figure 2:**
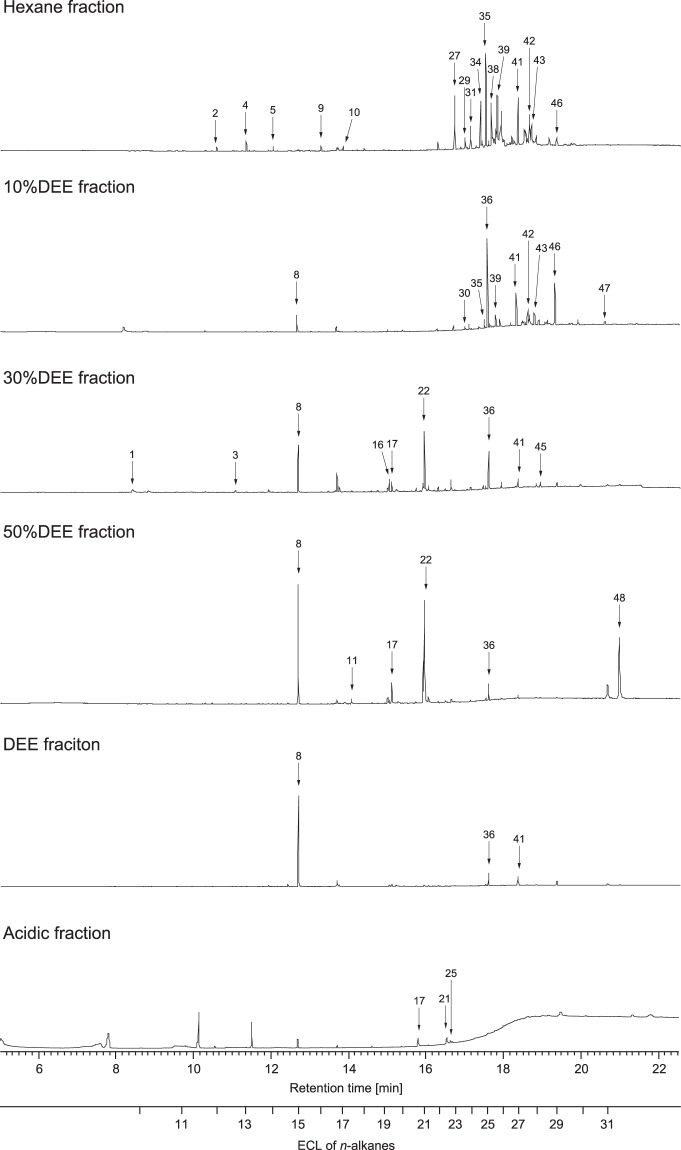
Representative gas chromatographs of the hexane, 10% DEE, 30% DEE, 50% DEE, DEE, and acid fractions. Chromatographs were derived from Colony B. ECL: equivalent of carbon length. Numbers associated with arrows indicate different compounds. Peaks without arrows mean unidentified compounds. Compounds 10, 17, 22, 35, 41, and 48 (2PhC11, PA, tVA, C25, C27, and Ch, respectively) constitute aggregation pheromone components (see Supplementary Fig. S1 for mass spectra of these six compounds). For compound names, see Supplementary Table S1.

To identify candidate pheromone components, the neutral and acidic fractions from four colonies were analysed using gas chromatography-mass spectrometry (GC-MS). In total, 29 compounds were commonly detected in all fractions from all colonies (Fig. 2, and Supplementary Table S1 and Dataset).

The hexane fractions contained hydrocarbons, including nine straight-chain hydrocarbons [*n*-dodecane, *n*-tridecane, *n*-tetradecane, *n*-hexadecane, *n*-tricosane (C23), *n*-tetracosane, *n*-pentacosane (C25), *n*-heptacosane (C27), and *n*-nonacosane], 2-phenylundecane (2PhC11), and six methyl-branched long-chain alkanes. All straight-chain hydrocarbons and 2PhC11 were identified by comparing retention times and mass spectra with commercial standards of *n*-alkanes and a synthesised standard of (±)-2PhC11 (see Supplementary Text S1). Although 2PhC11 is an optically active aromatic compound, enantioselective analysis indicated that the hexane fractions contained both (+)-(*S*)-2PhC11 and (−)-(*R*)-2PhC11 in a relative abundance ratio of approximately 2:1 (see Supplementary Text S2).

The 10% DEE fractions also contained six hydrocarbons, including C25 and C27. Importantly, 10% DEE fractions contained butylated hydroxytoluene and bis(2-ethylhexyl) adipate; however, butylated hydroxytoluene is a stabiliser contained in the diethyl ether solvent, while bis(2-ethylhexyl) adipate was assumed to be a contaminant. We therefore concluded that the aggregation activity of the 10% DEE fraction was explained by the presence of long-chain hydrocarbons (i.e., C25 and C27).

The 50% DEE fractions contained myristic acid (MA), vaccenic acid, palmitic acid (PA), and cholesterol (Ch), while the acid fractions contained oleic acid (OA) and PA (Fig. 2 and Supplementary Dataset). Comparative GC analyses of methyl ester derivatives identified the vaccenic acid as the *trans*-isomer (tVA) (*cis*-isomer: *t*_R_ = 17.94 min, *trans*-isomer: *t*_R_ = 17.25 min, natural vaccenic acid from 50% DEE fractions: *t*_R_ = 17.15 min). Collectively, these results suggested that the aggregation pheromone comprised straight short-chain hydrocarbons, an aromatic hydrocarbon, long-chain hydrocarbons, fatty acids, and/or cholesterol.

### Pheromone components and dose-dependent activity

We performed bioassays using multiple steps to identify candidate aggregation pheromone compounds. This process indicated that, at minimum, the components of the aggregation pheromone of *R. speratus* included (+)- and (−)-2PhC11, C25, C27, tVA, PA, and Ch; the optimal mixture ratio to induce worker attraction was (+)-2PhC11: (−)-2PhC11: C25: C27: tVA: PA: Ch = 0.3:0.3:13.4:8.6:40:200:11.6, respectively (see Supplementary Text S3 for process details).

**Figure 3:**
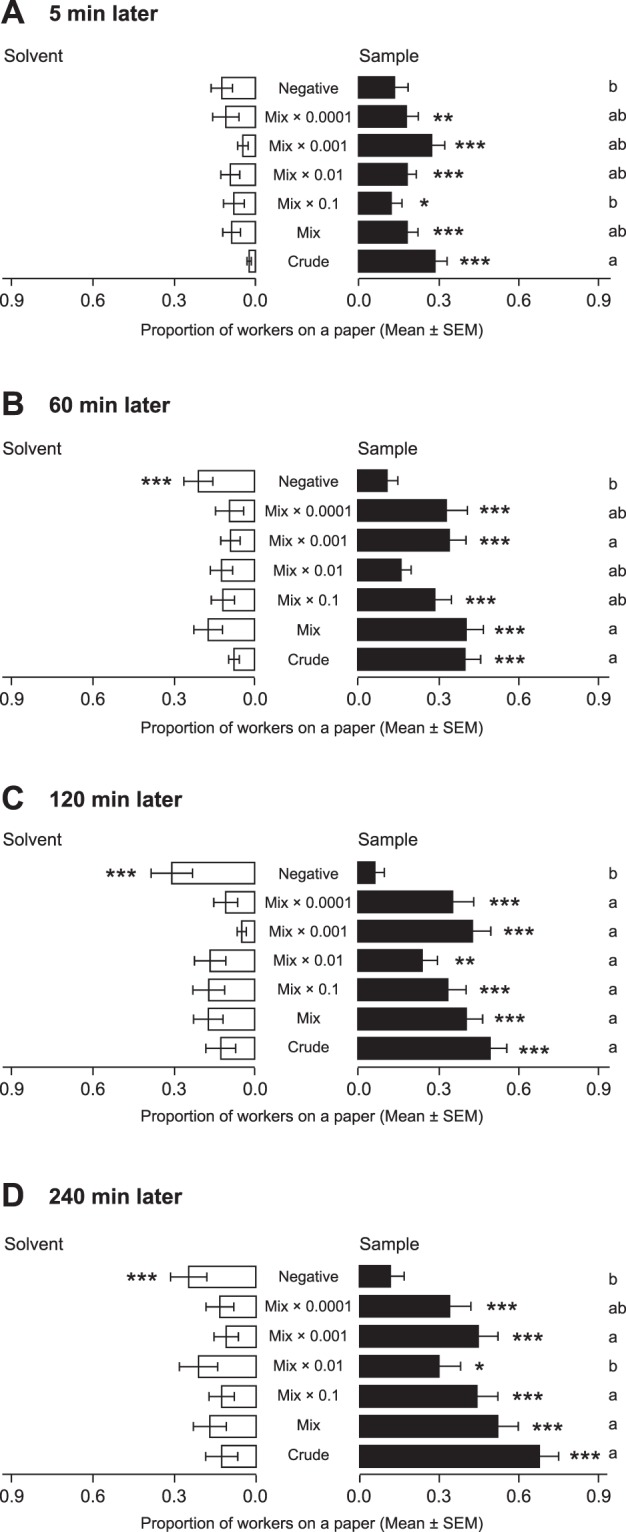
Dose responses of artificial aggregation pheromone after 5 min (**A**), 60 min (**B**), 120 min (**C**), and 240 min (**D**). ‘Mix’ was a mixture of six pheromone compounds [(±)-2PhC11: C25: C27: tVA: PA: Ch = 60:1340:860:4000:20000:1160 (unit: ng)]. ‘Mix × 0.1’, ‘Mix × 0.01’, ‘Mix × 0.001’, and ‘Mix × 0.0001’ indicate 10 to 10,000-fold dilutions of the Mix solution, respectively. For details of bar plots, see Fig. 1. Each treatment had 20 replicates (10 replicates × 2 colonies). Asterisks indicate significant differences between total numbers of workers on sample papers and total numbers of workers on solvent papers (binomial test with Bonferroni correction, * = *P* < 0.05, ** = *P* < 0.01, *** = *P* < 0.001). Different alphabets indicate significant differences in the mean of proportion of workers on sample papers among treatments (GLMM followed by Tukey HSD test, *P* < 0.05). (**A**) Mix solutions significantly attracted workers, as did the crude extract (binomial test with Bonferroni correction, *P* < 0.05). (**B–D**) All doses of Mix solutions (excluding Mix × 0.01) and the crude extract continued to show significant aggregation activity throughout the experimental period (binomial test with Bonferroni correction, *P* < 0.05).

To test the dose-dependency of the artificial aggregation pheromone and the duration of the arresting effect at various doses, we presented the Mix solution [(±)-2PhC11: C25: C27: tVA: PA: Ch = 60:1340:860:4000:20000:1160] and its 10 to 10,000 times diluted solutions to workers. We then compared the proportions of workers on sample papers among doses after 5, 60, 120, and 240 min of exposure. After 5 min, all workers were attracted by all concentrations and by the crude extract (binomial test with Bonferroni correction, *P* < 0.05, Fig. 3A), although the attraction level of the 10 times diluted solution seemed to be lower than other solutions (GLMM followed by Tukey HSD test, *P* < 0.05). On and after 60 min of exposure, workers continue to aggregate to all solutions, excluding Mix × 0.01, as well as to the crude extract (binomial test with Bonferroni correction, *P* < 0.05, Fig. 3B–D). Mix and Mix × 0.001 continued to show higher aggregation activities at later time periods (GLMM followed by Tukey HSD test, *P* < 0.05). Furthermore, in all doses and the crude extract, the mean proportions of workers on sample papers increased with the lapse of time (GLMM followed by multiple pairwise-comparisons of interaction between treatment and time with Bonferroni correction: *P* < 0.05), whereas the mean proportion of workers on solvent papers did not increase. These results indicated that the aggregation pheromone we identified had both attractant and arrestant activity, given that the pheromone continued to attract and arrest workers over time. In addition, in the crude extract, Mix and its diluted solutions treatments, workers kept to stay at the same papers after attracted by the paper. But in the negative control treatment, workers began to wander inside the dish or to move to another paper even after gathering at either of papers.

### Discharge of aggregation pheromone from worker’s body

The above chemical analyses and bioassays demonstrated that the inside of worker’s body includes 2PhC11, C25, C27, tVA, PA, and Ch, and that a mixuture of the six compounds shows worker attractant and arrestant activities. If the six compounds are actually emited from workers as pheromone components, they should adhere to termite’s nest material. To confirm whether the pheromone components are contained in the nest materials, shelter papers (the papers which are placed in breeding container with workers for 24 h) were extracted and analysed by GC-MS analysis. As a result, the shelter paper extract include all pheromone components (Supplementary Fig. S2 and Dataset), suggesting that workers emitted these six compounds on the outside of their bodies.

## Discussion

Aggregation behaviour is a hallmark of social insects. However, understanding of the proximate mechanisms that attract and arrest nestmates remains poor, particularly for termites. We demonstrated that the Japanese subterranean termite *R. speratus* uses an aggregation pheromone that attracts foraging workers and induces arrest at the pheromone source. The pheromone consists of six compounds: an aromatic compound (2PhC11), two straight-chain alkanes (C25 and C27), saturated and unsaturated carboxylic acids (PA and tVA, respectively), and Ch. When the worker crude extracts were fractionated (Fig. 1A), 2PhC11 was included in hexane fraction, C25 and C27 were in both hexane and 10%DEE fractions, tVA and Ch were in 50%DEE fraction, and PA was in 50%DEE and acidic fractions (Fig. 2 and Dataset). Because hexane, 10%DEE, 50%DEE, and acidic fractions have an attractant activity (Fig. 1B), the activity of hexane and 10%DEE fractions can be explained by 2PhC11, C25, and C27, and that of 50%DEE and acidic fractions can be explained by tVA, PA, and Ch. However, the attractant activity was lower in these fractions than the crude extract (Fig. 1B). These results suggest that each pheromone component can individually gather workers in a short time by attracting workers at a distance or stopping passing workers, but all six compounds presumably act synergistically to enhance attractant activity. Also, a test of the dose-dependency and the duration of arresting effect revealed that the number of aggregated workers gradually increased with time in all doses of the mixture of the six compounds (Fig. 3). This indicates that the mixture remains on the paper for a long time and continue to attract and arrest passing workers. On the other hand, the number of workers on solvent papers increased and then decreased with time in the negative treatment (Fig. 3). If there is no attractant agent in the dish, it should be determined by chance which papers workers aggregate at. But in negative treatment, we observed that even if the same number of workers gather at each paper for the first 5 min, all workers eventually gathered at the same paper later, and then some workers started to wander again. Probably, in many dishes, workers occasionally gathered at the solvent papers 60–120 min after starting the experiment, and then they started to move to the sample papers 240 min after. Therefore, we estimated that, if there is no attractant/arrestant agent, *R. speratus* workers repeat moving and staying, but if there is an attractant/arrestant agent in a certain place, workers gather at the source of the agent from the beginning and continue to stay there. From the above, the aggregation pheromone of *R. speratus* workers is also considered to have a strong arrestant activity.

Aggregation pheromones have evolved in various insect groups, but in many cases, the pheromone components generally consist of multiple compounds mainly including volatile aldehydes, alcohols, esters, ketones, aromatic compounds, and terpenoids. For example, in a locust, adult locusts use aromatic compounds, and nymphal locusts use volatile aldehydes, volatile carboxylic acids, aromatic compounds, and indole^23^. Stink bugs use sesquiterpenoids or esters^24–26^, a thrip use a blend of esters^27^, and bed bugs use a mixture of volatile sulfur-containing compounds, aldehydes and a ketone^5^. In bees and wasps, *Colletes* bees use volatile terpenoids^6^, a wood wasp use a volatile alcohol^28^, and parasitoid wasps use volatile ketones and spiroacetals^29,30^. Moths use volatile ketones, aldehydes, monoterpene, and alcohols^31,32^. Beetles use various types of compounds as aggregation pheromones depending on taxonomic groups; red flour beetles use a volatile aldehyde^33^, bark beetles mainly use terpenoids^34^, and longhorn beetles use mixtures of volatile ketones, alcohols, aromatic compounds, and terpenoids^35–38^. On the other hand, some insects use not only volatile compounds but also non-volatile compounds as aggregation pheromones. For example, *Drosophila* flies use esters, ketones, and long-chain hydrocarbons^39–42^. Although German cockroach is the first species that the presence of aggregation pheromone is suggested^43^, the identification of this pheromone remained to be discussed. But a recent study revealed that German cockroaches use volatile carboxylic acid or non-volatile fatty acids as aggregation agents, and the components of the agent vary a little depending on the cockroach populations^44^. In termites, our results demonstrated that *R. speratus* workers use an aromatic compound, long-chain hydrocarbons, and fatty acids for their aggregation pheromone. Considering that the compounds used for insect pheromones (alcohols, aldehydes, esters, hydrocarbons, carboxylic acids, and terpenes) are generally biosynthesised via lipid metabolic pathways^45–47^, it is suggested that basic components of insect aggregation pheromones are synthesised from lipid derivatives, and that adding idiosyncratic compounds (e.g., compounds having unique structures, other compound groups such as aromatic compounds) to a preexisting aggregation pheromone may contribute to differentiating pheromone components from those of other species.

Cuticular hydrocarbons (CHCs) play an important role in nestmate and caste recognition in social insects^48^. The CHCs of worker *Reticulitermes* termites include straight (or branched) alkanes and alkenes with >20 carbon molecules; these may act as cues in species and nestmate recognition, as demonstrated in ants^49^. The GC-MS analyses detected straight-chain and methyl-branched alkanes bearing 23–31 carbon molecules from the extracts of *R. speratus* workers; C25 and C27 were the two most abundant compounds among those found in CHCs among workers (Fig. 2 and Supplementary Table S1). Bioassays demonstrated that all CHC components are not required when workers aggregate at the source of aggregation pheromone (Fig. 3). This implies that other minor hydrocarbons may not contribute to worker aggregation.

The presence of Ch in the aggregation pheromone is of considerable interest. Steroids act as precursors of ecdysteroid hormones in insects, which regulate molting; however, insects typically have no ability to synthesise steroids *de novo*^50^. Therefore, insects must obtain steroids through predation of other organisms, or from gut symbionts^51–53^. Because there are no study about biosynthetic and metabolic process of steroids in *R. speratus*, the origin of Ch is still unknown. There are two possibilities: 1) workers get Ch from dead wood, or 2) worker’s gut symbionts produce Ch (or its derivatives), but further studies will be needed.

Pheromones are involved in most social activities observed in social insects^54^, and aggregation pheromones regulate a variety of collective behaviours^55,56^. Our results demonstrated that *R. speratus* workers produce a multicomponent aggregation pheromone by combining an aromatic hydrocarbon and lipids with major CHC components. This suggests that foraging workers use the aggregation pheromone to inform other workers of new locations that are suitable for colonisation; moreover, rapid aggregation contributes to efficient feeding and colony expansion. Our work has provided new insight into the chemical communication systems underlying social assembly in termites.

## Materials and Methods

### Termite collection

*R. speratus* colonies were collected from secondary forests in Kyoto and Shiga Prefectures, Japan, from April 2018 to April 2020 (colonies A–L). Colony E was collected from Kutsuki, Takashima City, Shiga Prefecture. Colonies A and B were collected in Kita-ku, Kyoto City, Kyoto Prefecture. Colony H, I, and J were collected in Amarube-cho, Kameoka City, Kyoto Prefecture. The remaining colonies were collected in Ukyo-ku, Kyoto City, Kyoto Prefecture.

### Ethics

No specific permits were required for the described field activities. No specific permissions were required to access or sample termite colonies, as these colonies were collected from unprotected public lands. This study did not involve endangered or protected species.

### Extraction and isolation

To extract chemical compounds from the bodies of termite workers, 2,000 individuals per colony were extracted in a 100 mL glass vial containing 10 mL *n*-hexane (FUJIFILM Wako Pure Chemical Corporation, Osaka, Japan) for 24 h. Four samples were derived from four colonies (A, B, K, and L). The hexane crude extracts (equivalent to 200 workers/mL) were transferred into new glass centrifuge tubes for further processing and were preserved in a freezer (−30 °C) until use in further analyses.

Fractionation of the crude worker extract was performed using the following procedure (Fig. 1A). First, 2 mL of crude extract was extracted with 1 M hydrogen chloride (HCl, 3 × 4 mL) and then fractionated into hexane and aqueous layers. The aqueous layer was alkalised (pH 10) with 1 M sodium hydroxide (NaOH, 18 mL), then extracted with hexane (3 × 4 mL). This extract was concentrated without desiccation using a rotary evaporator to obtain the basic fraction (2 mL). The hexane layer was added to 2 mL of hexane, extracted with 1 M NaOH (3 × 4 mL), and then fractionated into a 4-mL hexane layer (hereafter, the neutral fraction) and an aqueous layer. This aqueous layer was acidified (pH 1) with 1 M HCl (18 mL) and extracted with hexane (3 × 4 mL). This extract was then concentrated without desiccation using a rotary evaporator to obtain the acid fraction (2 mL). The neutral fraction (1 mL) was chromatographed on a silica gel column (0.5 g, Wako gel C-200; FUJIFILM Wako Pure Chemical Corporation). The column was successively eluted with hexane, three concentrations of DEE (FUJIFILM Wako Pure Chemical Corporation) in hexane (2 mL each of 10% DEE, 30% DEE, and 50% DEE), and DEE (2 mL). Resulting 100-μL aliquots from each fraction were concentrated to 10-μL aliquots using a gentle nitrogen steam and then subjected to GC-MS analyses for identification. The remainder of each fraction was preserved in a freezer (−30 °C) for later use in bioassays and further chemical analyses.

### Preparation of shelter paper extracts

To elucidate substances excreted by worker termites, we used a shelter paper approach. Groups of 200 workers were placed in 35-mm plastic dishes that were lined with 30-mm filter papers (Advantec No. 2; Toyo Roshi Kaisha, Ltd, Tokyo, Japan) (Fig. S2). We prepared 26 and 18 dishes from the colonies C and F, respectively, because total number of workers included in the nest wood was different between colonies. The papers were moistened with 150 μL of distilled water and the dishes were incubated at 25 °C. Shelter papers were collected from each dish every 24 h for 3 days, then preserved in a freezer at −20 °C for use in later analyses.

Chemical compounds were obtained from shelter papers by extraction with hexane in a 200-mL glass jar for 24 h (colony C: 78 papers/78 mL; colony F: 54 papers/54 mL). The resulting crude extract (500 μL) was chromatographed on a silica gel column (0.5 g, Wako gel C-200, FUJIFILM Wako Pure Chemical Corporation). The column was successively eluted with hexane, three concentrations of DEE in hexane (1.5 mL each of 10% DEE, 30% DEE, 50% DEE), and DEE (1.5 mL). All fractions (1.5 mL each) were concentrated to 100-μL aliquots using a gentle nitrogen steam. Aliquots were then subjected to GC-MS analyses for identification.

### GC-MS analysis

GC-MS analyses were performed on a JMS-Q1500GC (JEOL Ltd., Tokyo, Japan) combined with an Agilent Technologies 7890B GC system (Agilent Technologies, Santa Clara, CA, USA) equipped with a DB-1MS column (30 m × 250 μm × 0.25 μm, Agilent Technologies). Column temperatures were set at 50 °C for 5 min, followed by 20 °C for 1 min, and then 300 °C for 5 min. A 1-μL hexane solution was then injected into each sample. The injector used was in splitless mode, with helium as the carrier gas (1.2 mL/min), and the injection port temperature was maintained at 250 °C. MS data were obtained under the following conditions: 50 μA ionisation current, 70 eV ionisation energy, 2 kV accelerating voltage, and a 40–500 *m/z* scan range. Both GC and MS systems were controlled using a msPrimo System Controller ver. 1.06 (JEOL Ltd.). We used the software Escrime ver. 2.04 (JEOL Ltd., https://www.jeol.co.jp/en/products/detail/JMS-Q1500GC.html) for data analysis. Candidate compounds were predicted from the Mass Spectral Library (NIST11). Compounds were identified according to retention time and mass spectra, in comparison with commercial standards and synthesised racemic 2-phenylundecane standards (see Text S1).

### Methyl esterification of vaccenic acid

To determine the geometrical isomer of vaccenic acid derived from worker extracts, a fatty acid methyl ester was prepared prior to GC analysis. The 50% DEE fractions (each equivalent to 0.5 workers), and 1 μg each of authentic *cis*- and *trans*-vaccenic acid standards (Funakoshi Co., Ltd., Tokyo, Japan) were individually resuspended in 500 μL of 5% HCl methanol solution (FUJIFILM Wako Pure Chemical Corporation) in a 10-mL glass centrifuge tube. Each tube was then heated at 50 °C for 30 min. After the tube had cooled, the product was extracted with hexane (500 μL) three times. Combined hexane layers (1.5 mL) were concentrated using a gentle nitrogen steam (15 μL), then subjected to GC analyses. GC analyses were performed on a GC-2014 (Shimadzu Corporation, Kyoto, Japan) with a flame ionisation detector (220 °C). Fractions (1 μL each) were injected in splitless mode. The injection port temperature was 210 °C. The column used was an HP-88 (30 m × 250 μm × 0.25 μm, Agilent Technologies). The carrier gas was helium, with a flow rate of 20 mL/min. The oven was programmed as follows: 1 min at 120 °C, followed by 10 min at 175 °C, and then 1 min at 210 °C, in accordance with the manufacturer’s recommended protocol (Agilent Technologies Inc., 2014)^57^. The geometrical isomer of methyl vaccenate was identified by comparing its retention time with those of the methyl-esterified commercial standards.

### Bioassay

Prior their use in bioassays, approximately 2,000 termites were placed in a 140-mm plastic dish with a moistened filter paper (90 mm in diameter) in darkness at 25 °C for 2 days. Following acclimation, 30 workers each were placed into 90-mm plastic dishes that contained two pieces of filter paper (30 mm in diameter). Filter papers were placed on opposite sides of the dishes, which had polished surfaces. The ‘sample’ paper had an extract (or authentic standard) solution applied to it, whereas the ‘solvent’ paper had a solvent applied. Both papers were dried for approximately 5 min to remove solvents before the dish was covered. To record the number of workers on the sample and solvent papers, vertical photographs of each dish were taken using a digital camera (EOS Kiss Digital N + EF 50 mm f/2.5 Macro, Canon, Tokyo, Japan). Numbers of workers present on each paper were recorded after 5 min in all behavioural experiments, excluding those otherwise specified. All bioassays included 10 replications per colony per treatment, and workers from two colonies were used in each treatment.

Aggregation pheromone components were identified and the optimal ratio and dose were determined through the following three steps:

1. Comparison of attraction activities among fractions
  To examine aggregation activity among fractions (acidic, neutral, basic, hexane, 10% DEE, 30% DEE, 50% DEE, and DEE), workers from two colonies (C and D) were used. Worker crude extract derived from Colony A was used as a positive control. In the negative control treatment, sample and solvent papers were impregnated with hexane. For each replicate, a fraction solution (equivalent to 1 worker) was added to the sample paper (negative control: 10 μL; crude: 5 μL; acid and basic fraction: 5 μL; neutral fraction: 10 μL; hexane, 10% DEE, 30% DEE, 50% DEE, and DEE fractions: 20 μL). The same amount of solvent was added to the solvent paper in each treatment.
2. Determination of pheromone components and effective mixture ratios.
  To determine pheromone components and their optimal ratio, bioassays were performed in multiple steps. First, candidate compounds were identified in the hexane, 10% DEE, 50% DEE, and acidic fractions. Attractant activities of various combinations of the candidate compounds were tested using authentic standards. Various mixture ratios of these compounds were then tested. Experimental process details are provided in Text S3.
3. Dose response of artificial aggregation pheromone

The activity of the Mix solution [(±)-2PhC11: C25: C27: tVA: PA: Ch = 60:1340:860:4000:20000:1160 (unit: ng per 10-μL hexane)] was compared with the activities of four dilutions (Mix × 0.1, Mix × 0.01, Mix × 0.001, and Mix × 0.0001). Workers from Colonies I and J were used in these experiments. In each replicate of each treatment, excluding the positive control, 10 μL of solution were added to the sample paper, and the same volume of hexane was added to the solvent paper. In the positive control, 5 μL of crude extract derived from colony I (equivalent to 1 worker) were added to the sample paper. In the negative control treatment, hexane was applied to both filter papers. The numbers of workers present on the sample and solvent papers were recorded after 5, 60, 120, and 240 min to confirm the arrestant activities of pheromone components.

### Statistical analyses

Statistical analyses and graphical visualisations were performed in R software ver. 3.3.3^58^. For the behavioural assessment, the total numbers of workers on sample papers were compared with the total numbers of workers on solvent papers in each treatment, using a binomial test with a Bonferroni correction. All replicates were pooled for each treatment. Also, we compared the mean proportion of workers on sample papers among treatments, using a generalised linear mixed model (GLMM) followed by Tukey HSD test (*P* < 0.05). The proportion of workers on sample papers was regarded as the response variable, assuming a binomial distribution. Treatments were used as an explanatory variable and colonies and replicates were treated as randam effects.

For the bioassay experiments used to test dose-dependency and arrestant activity, the proportions of workers on sample papers were compared among treatments and time exposure periods using a GLMM approach. The proportion of workers on sample or solvent papers was regarded as the response variable, assuming a binomial distribution. Interactions between treatments and exposure times were used as explanatory variables and colonies and replicates were treated as random effects. Multiple pairwise-comparisons using a χ^2^ test were performed for post hoc comparisons interaction terms and a sequential Bonferroni correction was applied to evaluate pairwise significance.

## Supplementary information

Supplementary Information.

Supplementary Information2.
