# Supplementary material for: Chemical identification of an aggregation pheromone in the termite Reticulitermes speratus
Source: Sci Rep. 2020 May 4;10:7424. doi: 10.1038/s41598-020-64388-4 (PMC7198579; doi:10.1038/s41598-020-64388-4)
Supplement: Supplementary file 1 — Supplementary Information. [file 41598_2020_64388_MOESM1_ESM.pdf]

Supplementary information

Chemical identification of an aggregation pheromone in the termite *Reticulitermes speratus*

Yuki Mitaka[1]*, Shigeru Matsuyama[2], Nobuaki Mizumoto[3], Kenji Matsuura[4], and Toshiharu Akino[1]

[1] Applied Entomology Laboratory, Department of Bioresource Field Sciences, Kyoto Institute of Technology, Kyoto, 616-8354, Japan.

[2] Life Sciences and Bioengineering, Graduate School of Life and Environmental Sciences, University of Tsukuba, Ibaraki, 305-8572, Japan

[3] School of Life Sciences, Arizona State University, AZ, 85287-9425, USA

[4] Laboratory of Insect Ecology, Division of Applied Biosciences, Graduate School of Agriculture, Kyoto University, Kyoto, 606-8502, Japan.

* Corresponding author:

Yuki Mitaka (Email: ymitaka02@gmail.com)

Contents

Supplementary figures 2

Table S1. List of compounds in fractions of worker extracts 4

Text S1. Synthesis of racemic 2-phenylundecane 6

Text S2. Synthesis of enantiomers of 2-phenylundecane 10

Text S3. Identification process of aggregation pheromone 17

Supplementary references 30

Supplementary figures

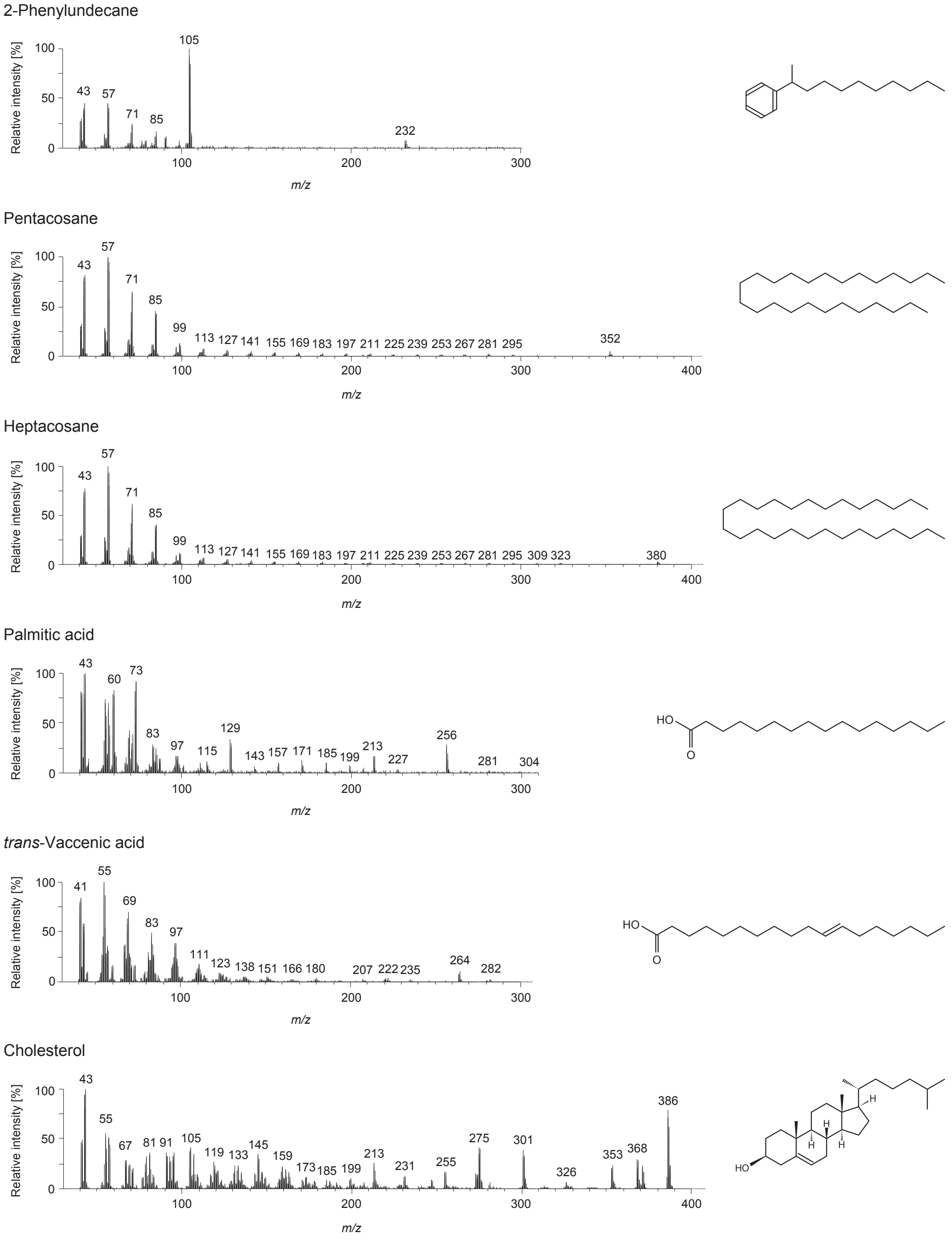

Figure S1. Representative mass spectra of the aggregation pheromone components contained in a worker extract (colony A).

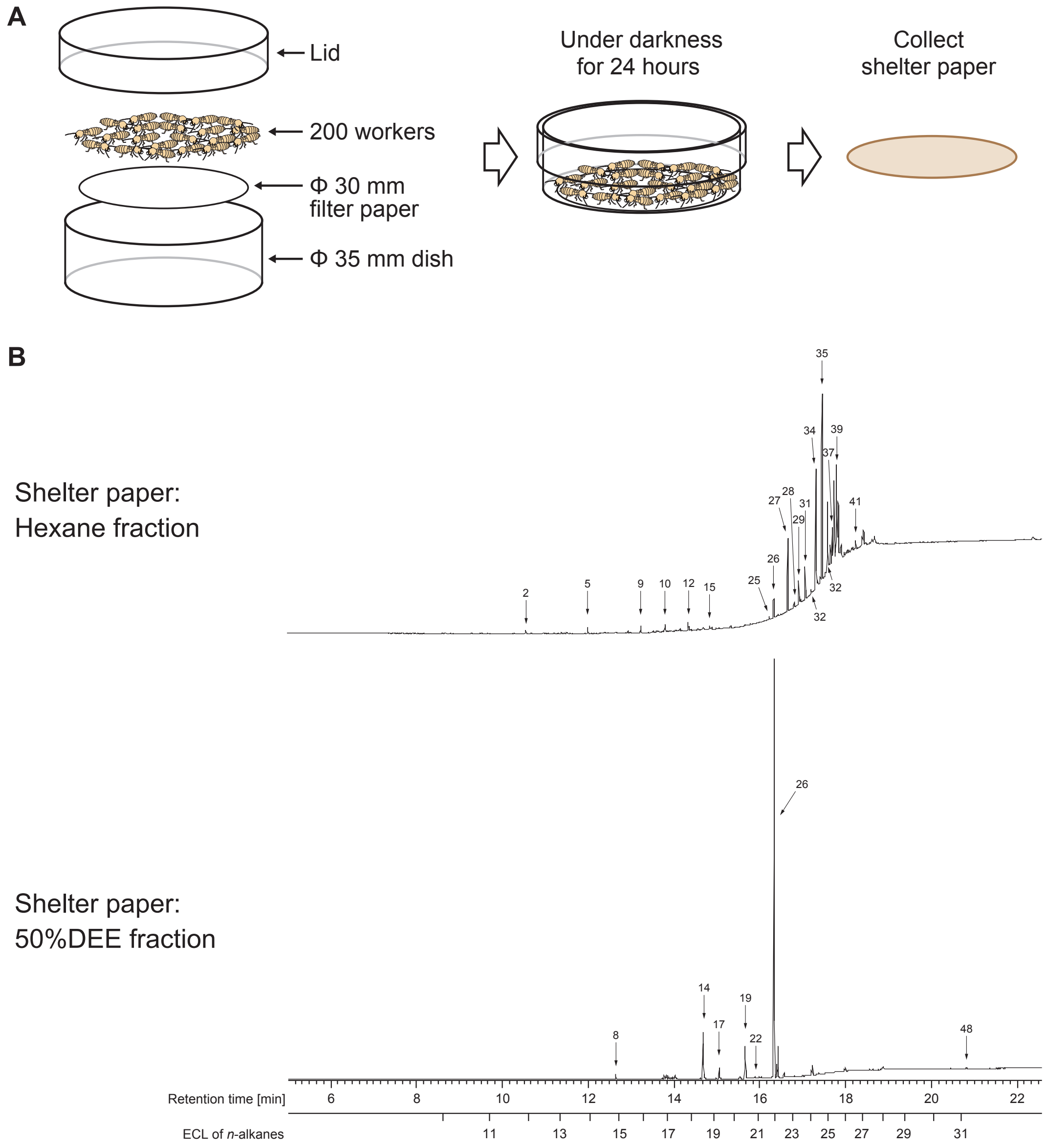

Figure S2. Chemical analysis of shelter paper extract. (A) Schematic drawing of rearing dish for collecting shelter papers. (B) Representative gas chromatographs of hexane and 50% DEE fractions of shelter paper extract from the same colony (colony C). The numbers indicated by arrows indicate different compounds. Compound 10, 17, 22, 35, 41 and 48 (2PhC11, PA, tVA, C25, C27 and cholesterol, respectively) are aggregation pheromone components. For all compound names, see Results section or Table S1.

Table S1. Compound list.

Peak No.	Compound name	Kovats index
1	3-Octanol	982
2	*n*-Dodecane*	1200
3	Indole	1263
4	*n*-Tridecane*	1300
5	*n*-Tetradecane*	1400
6	Neryl (*S*)-2-methylbutanoate	1431
7	1-Dodecanol	1449
8	Butylated hydroxytoluene	1497
9	*n*-Hexadecane*	1600
10	**2-Phenylundecane***	1700
11	Myristic acid*	1741
12	*n*-Octadecane*	1800
13	1-*o*-Octanoyl-xylitol	1833
14	1-Hexadecanol	1850
15	*n*-Nonadecane*	1900
16	Dibutyl phthalate	1933
17	**Palmitic acid***	1947
18	*n*-Eicosane*	2000
19	1-Octadecanol	2048
20	*n*-Heneicosane*	2100
21	Oleic acid*	2103
22	***trans*-Vaccenic acid***	2124
23	Butyl citrate	2132
24	Stearic acid*	2146
25	*n*-Docosane*	2200

Peak No.	Compound name	Kovats index
26	Tributyl acetylcitrate	2228
27	*n*-Tricosane*	2300
28	11-Methyltricosane	2308
29	2-Methyltricosane	2371
30	Bis(2-ethylhexyl) adipate	2375
31	*n*-Tetracosane*	2400
32	13-Methylpentacosane	2408
33	1,3-Diaceto-2-myristin	2416
34	2-Methyltetracosane	2471
35	***n*-Pentacosane***	2500
36	Bis(2-ethylhexyl) phthalate	2516
37	2-Methylpentacosane	2528
38	11-Methylpentacosane	2540
39	3-Methylpentacosane	2577
40	2,3-Bis(acetyloxy)propyl hexadecanoate	2601
41	***n*-Heptacosane***	2700
42	2-Methylheptacosane	2763
43	3-Methylheptacosane	2773
44	2-(Acetyloxy)-1-[(acetyloxy)methyl]ethyl octadecanoate	2793
45	Squalene	2819
46	*n*-Nonacosane*	2900
47	*n*-Hentriacontane*	3100
48	**Cholesterol***	3146

*: identified compound (Bold: aggregation pheromone components). For relative peak amount of each compound in each fraction of worker and shelter paper extracts, see ‘GC results’ sheet in Dataset.

Text S1. Synthesis of racemic 2-phenylundecane

Chemicals

Undecan-2-one and DMAP were purchased from Tokyo Chemical Industry Co., Ltd. Phenylmagnesium bromide was from Sigma-Aldrich Co. LLC. Methanesulfonyl chloride, triethylamine, 5% Pd/C, and silica gel (C-200) were obtained from FUJIFILM Wako Pure Chemical Corporation. All the dry solvents were 'Super Dehydrated' grade from FUJIFILM Wako Pure Chemical Corporation.

Chemical analyses

GC analyses were conducted on an HP6890 (Agilent Technologies) gas chromatograph equipped with a polar fused-silica capillary column DB-23 (Agilent Technologies, 30 m × 0.25 mm, 0.25 μm film thickness). Samples were injected at 250°C in the split mode (100:1). Helium was used as a carrier gas at 1 mL/min in the constant flow mode. Oven temperature was programmed from 110°C (held for 1 min) to 230°C at a rise of 10°C/min, and held for 5 min at 230°C. Eluents from the column were detected with a flame ionization detector (FID) at a temperature of 250°C. Chromatograms were analysed by ChemStation ver. A10.01 (Agilent Technologies).

Mass spectra were obtained by GC-MS using an HP6890N (Agilent Technologies) gas chromatograph connected to JEOL 600H mass spectrometer (JEOL). Samples were injected at 280°C in the split mode (100:1) into a nonpolar column DB-5MS (Agilent Technologies, 25 m × 0.25 mm, 0.25 μm film thickness) under a helium flow at 1 mL/min in the constant flow mode. Components in the samples were separated with an oven temperature program of 50°C (1 min held) to 320°C at 10°C/min (held for 2 min), introduced into the ionization chamber at 190°C and ionized at 70eV. Mass spectral data were collected in the scan mode (scan range: 40 – 600 amu), at a scan speed of 0.29 sec, analysed by a software TSS2000 (Shrader Analytical Consulting Laboratory, Inc.).

Infrared spectra were measured with an IR Report-100 (JASCO corporation).

Synthesis of racemic 2-phenylundecane

Racemic 2-phenylundecane was prepared by three steps starting from a Grignard reaction between phenylmagnesium bromide and undecan-2-one, followed by dehydration of the corresponding alcohol [4] and palladium catalysed hydrogenation of the product.

2-Phenylundecan-2-ol (**2**): An ice-cooled stirred solution of undecan-2-one (**1**, 1.70 g, 10 mmol) in dry ether (15 mL) was added dropwisely a solution of phenylmagnesium bromide (3M in ether, 3.3 mL, 9.9 mmol) via a syringe. Stirring was continued with cooling for 0.5 h, then at room temperature for 1 h before quenching. The reaction mixture was poured into an aqueous saturated solution of NH_4Cl, extracted three times with ether. Combined organic layers was washed successively with water, brine and dried over $MgSO_4$. After evaporation of the solvent, residual oil was chromatographed over silica gel to afford 1.93 g (7.8 mmol, 78% yield) of 2-phenylundecan-2-ol (**2**). GC-MS (DB-5MS: 17.25 min), m/z (%): 230 (14, $M^+ - H_2O$), 132 (7), 131 (51), 129 (7), 121 (34), 119 (10), 118 (100), 117 (17), 115 (7), 105 (10), 91 (17). IR (neat, NaCl, cm^{-1}): 3425 (m), 3080 (w), 3060 (w), 3030 (w), 2940 (s), 2860 (s), 1600 (w), 1495 (w), 1375 (m), 1120 (m), 760 (s), 700 (s).

2-Phenylundecenes (**3**): A stirred mixture of 2-phenylundecan-2-ol (**2**, 1.93 g, 7.8 mmol), triethylamine (2.37 g, 23.4 mmol, 3.0 eq), and *N,N*'-dimethyl-4-aminopyridine (DMAP, 0.04 g, 0.3 mmol, 4 mol%) in dry CH_2Cl_2 (40 mL) was cooled with an ice-water bath. To the solution, methanesulfonyl chloride (MsCl, 1.34 g, 11.7 mmol, 1.5 eq) was added dropwisely via a syringe. The mixture was stirred with cooling for 0.5 h, at room temperature for 2 h, then poured into ice-water. The product was extracted with hexane, washed with water, brine and dried over $MgSO_4$. The solvent was evaporated to give an oil (1.81 g) which was chromatographed over silica gel and eluted with hexane to yield 1.41 g (6.1 mmol, 78% yield) of 2-phenylundecenes (**3**) as a mixture of isomers. This product gave three peaks by gas chromatography on both nonpolar (DB-5MS) and polar (DB-23) columns, and was subjected to hydrogenation without further purification. GC-MS (DB-

5MS: major peak at 16.62 min), m/z (%): 230 (26, M^+), 132 (13), 131 (100), 129 (11), 128 (6), 119 (10), 118 (95), 117 (15), 116 (8), 115 (9), 105 (13), 91 (25). IR (neat, NaCl, cm^{-1}): 3080 (w), 3060 (w), 3030 (w), 2940 (s), 2860 (s), 1600 (w), 1495 (w), 760 (s), 700 (s).

2-Phenylundecane (**4**): A solution of 2-phenylundecenes (**3**) in ethyl acetate (10 mL) was mixed with 0.5 g of 5% Pd/C, and hydrogenated using a hydrogen balloon for 2 h at room temperature. The mixture was filtered through a pad of celite. The filtrate was evaporated to leave a crude oil which was passed through a silica gel column eluting with hexane to give racemic 2-phenylundecane (**4**, 1.39 g, 6.0 mmol, overall yield: 60%) as a single peak by gas chromatography on both DB-5MS and DB-23 columns. GC-MS (DB-5MS: 15.52 min), m/z (%): 232 (27, M^+), 106 (28), 105 (100), 104 (9), 103 (5), 91 (18), 77 (5), 41 (5). IR (neat, NaCl, cm^{-1}): 3080 (w), 3060 (w), 3030 (w), 2960 (m), 2930 (s), 2860 (s), 1600 (w), 1495 (m), 760 (s), 700 (s).

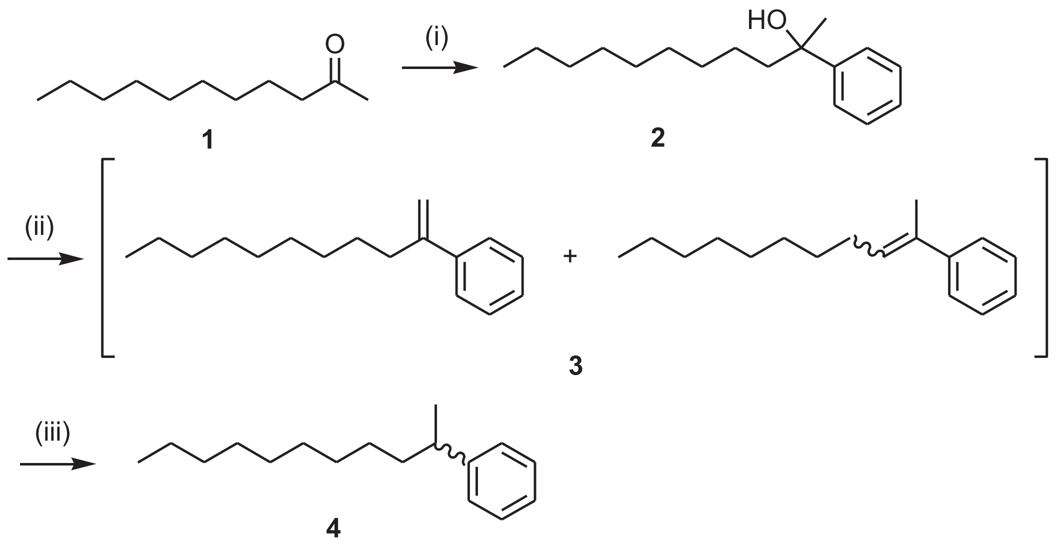

Figure S3. Preparation of racemic 2-phenylundecane. Reagents: (i) PhMgBr, Et_2O; (ii) MsCl, Et_3N, DMAP, CH_2Cl_2; (iii) 5% Pd/C, EtOAc

Synthesized (±)-2-phenylundecane

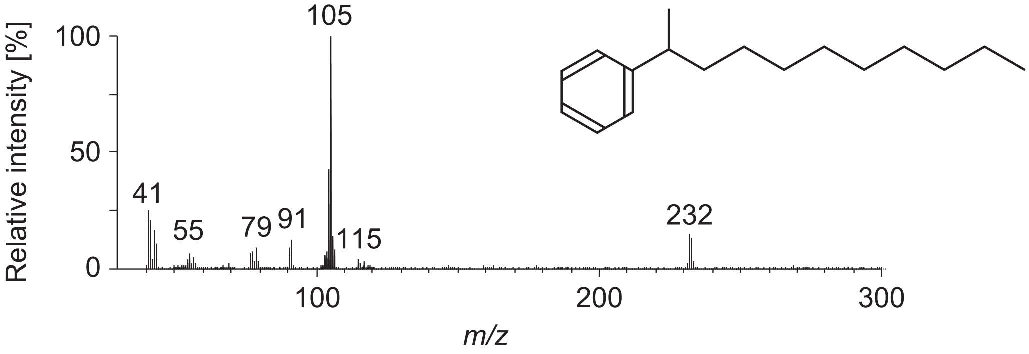

Figure S4. Mass spectrum of racemic 2-phenylundecane.

Text S2. Synthesis of enantiomers of 2-phenylundecane

Chemicals

(*S*)-(+)-2-Phenylpropanoic acid (> 98% ee), (*R*)-(−)-2-phenylpropanoic acids (> 98% ee), octylmagnesium bromide (1M, in THF) and carbon tetrabromide (CBr_4) were purchased from Tokyo Chemical Industry Co., Ltd. Dilithium tetrachlorocuprate(II) (Li_2CuCl_4) solution in THF was from Sigma-Aldrich Co. LLC. Lithium aluminum hydride ($LiAlH_4$), triphenylphosphine (PPh_3), and silica gel (Wako-gel, C-200) were obtained from FUJIFILM Wako Pure Chemical Corporation. All the dry solvents and 1-methyl-2-pyrrolidone (NMP) were 'Super Dehydrated' grade from FUJIFILM Wako Pure Chemical Corporation.

Chemical analyses

GC analyses were conducted on an HP6890 gas chromatograph equipped with a polar fused-silica capillary column DB-23. Samples were injected at 250°C in the split mode (100:1). Helium was used as a carrier gas at 1 mL/min in the constant flow mode. Oven temperature was programmed from 45°C (held for 1 min) to 230°C at a rise of 10°C/min, and held for 5.5 min at 230°C. Eluents from the column were detected with a flame ionization detector (FID) at a temperature of 250°C. Chromatograms were analysed by ChemStation ver. A10.01 (Agilent Technologies).

Mass spectra were obtained by GC-MS using an HP6890N gas chromatograph connected to JEOL 600H mass spectrometer. Samples were injected at 280°C in the split mode (100:1) into a nonpolar column DB-5MS (Agilent Technologies, 25 m × 0.25 mm, 0.25 μm film thickness) under a helium flow at 1 mL/min in the constant flow mode. Components in the samples were separated with an oven temperature program of 50°C (1 min held) to 320°C at 10°C/min (held for 2 min), introduced into the ionization chamber at 190°C and ionized at 70eV. Mass spectral data were collected in the scan mode (scan range: 40 – 600 amu), at a scan speed of 0.29 sec, analysed by a software TSS2000 (Shrader

Analytical Consulting Laboratory, Inc.).

Optical rotations were measured using a HORIBA high sensitive polarimeter SEPA-200. NMR spectra were recorded in $CDCl_3$ on a Bruker Avance-600 at 600MHz (1H-NMR) and 125MHz (13C-NMR). Chemical shifts are given relative to tetramethylsilane, and their multiplicities are abbreviated as m (multiplet), app t (apparent triplet), d (doublet), and s (singlet).

Synthesis of (*S*)-(+)-2-phenylundecane

(*S*)-(+)-2-Phenylundecane (**4a**) was prepared by cupper catalysed Grignard coupling between *n*-octylmagnesium bromide and (*R*)-(+)-1-bromo-2-phenylpropane (**3a**).

(*R*)-(+)-2-Phenylpropan-1-ol (**2a**): To an ice-cooled stirred suspension of $LiAlH_4$ (0.51 g, 13.3 mmol, 2.0 eq) in dry THF, (*R*)-(+)-2-phenylpropanoic acid (**1a**, 1.00 g, 6.67 mmol) in dry THF (5 mL) was added dropwise. The reaction mixture was stirred overnight, allowed to come to room temperature. While cooling with an ice-water bath, the reaction mixture was successively treated with water (0.51 mL), 15% NaOH aqueous solution (0.51 mL), and water (1.53 mL). Stirring was continued for 0.5 h with cooling, then at room temperature for an additional 0.5 h. Resulting suspension was suction filtered. Filtered cake was washed with ether, combined filtrate was dried over $MgSO_4$, and evaporated. The residual oil was chromatographed over silica gel to give 0.95 g of **2a** as a colourless oil. $[\alpha]_D^{20}$ +20.66 (*c* = 1.0, $CHCl_3$), lit [1] +13.1 (*c* 1.09, $CHCl_3$); GC-MS (DB-5MS: 9.03 min), m/z (%) 136 (15, M^+), 118 (7), 117 (9), 106 (28), 105 (100), 104 (6), 103 (12), 91 (11), 79 (15), 77 (12), 51 (5).

(*R*)-(+)-1-Bromo-2-phenylpropane (**3a**) [2]: To a stirred mixture of (*R*)-(+)-2-phenylpropan-1-ol (**2a**, 0.95 g, 7.0 mmol, 1.0 eq) and CBr_4 (2.92 g, 8.8 mmol, 1.25 eq) in dry THF (5 mL), PPh_3 (2.31 g, 8.8 mmol. 1.25 eq) was added portionwise. The mixture was stirred for 2 h, then poured into water and extracted with ether. Combined ether layers were washed with brine and dried over $MgSO_4$. After filtration and concentration, the residue was triturated with hexane and the resulting precipitation was filtered off. Silica gel

chromatography of the product yielded 1.45 g of **3a** as a colourless oil. $[\alpha]_D^{20}$ +18.46 (c = 1.0, $CHCl_3$), lit [1] +17.5 (c 0.95, EtOH); GC-MS (DB-5MS: 10.24 min), m/z (%) 200 (12, M^+), 198 (13, M^+), 119 (6), 118 (8), 1117 (9), 115 (5), 106 (9), 105 (100), 104 (8), 103 (7), 91 (14), 79 (5), 77(9).

(*S*)-(+)-2-Phenylundecane (**4a**): To a solution of (*R*)-1-bromo-2-phenylpropane (**3a**, 1.45g, 7.2 mmol) in dry THF (10 mL), Li_2CuCl_4 (0.1M THF, 2.2 mL, 0.22 mmol, 3 mol%) and NMP (2.8 mL, 28.8 mmol, 4.0 eq) were added via syringes. While cooling the mixture with an ice-water bath, *n*-octylmagnesium bromide in THF (1M, 8.0 mL, 8.0 mmol, 1.1 eq) was dropped via a syringe. The reaction mixture was stirred overnight, allowed to come to room temperature, then poured into ice-cooled aqueous NH_4Cl solution, and extracted with ether. Combined extracts were washed with aqueous of NH_4Cl, water, brine, and dried over $MgSO_4$. Evaporation of the solvent gave an oil which was chromatographed over silica gel to yield 0.33 g (1.42 mmol, overall 21% yield, 96% purity by GC) of the product as a colourless oil. For chemical analyses and bioassays, further purification was conducted to give 0.20 g of **4a** with 98.3% purity by GC. $[\alpha]_D^{20}$ +25.46 (c = 1.0, $CHCl_3$); GC-MS (DB-5MS: 15.89 min), m/z (%): 232 (15, M^+), 106 (13), 105 (100), 104 (5), 91 (10); ^{1}H-NMR (600MHz, $CDCl_3$) δ (ppm) 7.28 (2H, app t), 7.17 (3H, app t), 2.67 (1H, m), 1.56 (2H, m), 1.29-1.22 (16H, m), 1.15 (1H, m), 0.87 (3H, t, 6.0Hz); ^{13}C-NMR (150MHz, $CDCl_3$) δ (ppm); 148.0 (s), 128.2 (d), 128.2 (d), 127.0 (d), 127.0 (d), 125.7 (d), 39.9 (d), 38.5 (t), 31.9 (t), 29.7 (t), 29.6 (t), 29.6 (t), 29.3 (t), 27.7 (t), 22.7 (t), 22.3 (q), 14.1 (q).

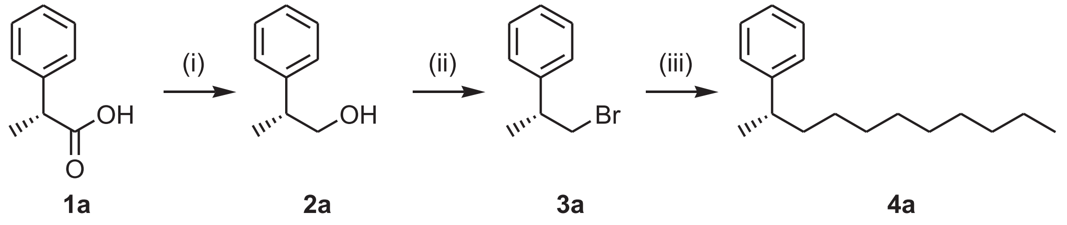

Figure S5. Preparation of (*S*)-(+)-2-phenylundecane. Reagents: (i) $LiAlH_4$, THF; (ii) CBr_4, PPh_3, THF; (iii) C_8MgBr, NMP, Li_2CuCl_4, THF.

Synthesis of (*R*)-(−)-2-phenylundecane

Synthesis of (*R*)-2-phenylundecane (**4b**) was prepared from (*S*)-1-bromo-2-phenylpropane in the same way as described for the preparation of (*S*)-isomer (**4a**).

(*S*)-(−)-2-Phenylpropan-1-ol (**2b**): In the same manner as described for the preparation of **2a**, (*S*)-(−)-2-phenylpropanoic acid (**1b**, 1.00 g, 6.67 mmol) was reduced by $LiAlH_4$ (0.51 g, 13.3 mmol, 2.0 eq) in dry THF to give **2b** (0.95 g) as a colourless oil. $[\alpha]_D^{20}$ −20.66 (c = 1.0, $CHCl_3$), lit[2)] −13.9 (c 1.22, $CHCl_3$); GC-MS (DB-5MS: 9.03 min), m/z (%): 136 (16, M^+), 118 (5), 117 (6), 106 (27), 105 (100), 104 (5), 103 (11), 91 (11), 79 (14), 77 (12).

(*S*)-(−)-1-Bromo-2-phenylpropane (**3b**): According to the procedure described for **3a**, (*S*)-(−)-2-phenylpropan-1-ol (**2b**, 0.95 g, 7.0 mmol, 1.0 eq) was treated with CBr_4 (2.92 g, 8.8 mmol, 1.25 eq) and PPh_3 (2.31 g, 8.8 mmol. 1.25 eq) in dry THF (5 mL) to yield **3b** (1.45 g) as a colourless oil. $[\alpha]_D^{20}$ −16.66 (c = 1.0, $CHCl_3$), lit [1] −17.8 (c 0.78, EtOH); GC-MS (DB-5MS: 10.25 min), m/z (%): 200 (11, M^+), 198 (13, M^+), 119 (5), 118 (6), 117 (9), 115 (5), 106 (9), 105 (100), 104 (8), 103 (7), 91 (12), 79 (6), 77 (7), 51 (5).

(*R*)-(−)-2-Phenylundecane (**4b**): (*S*)-(−)-1-Bromo-2-phenylpropane (**3b**, 1.45g, 7.2 mmol) was coupled with *n*-octylmagnesium bromide in the same manner as described for **4a** to afford **4b** (0.40 g, 1.42 mmol, overall 21% yield, 97.1% purity by GC) as a colourless oil. For chemical analyses and bioassays, further purification was conducted to give 0.13 g of **4b** with 98.5% purity by GC. $[\alpha]_D^{20}$ −23.26 (c = 1.0, $CHCl_3$); GC-MS (DB-5MS: 15.88 min), m/z (%): 232 (13, M^+), 106 (12), 105 (100), 104 (5), 91 (8); ^{1}H-NMR (600MHz, $CDCl_3$) δ (ppm); 7.28 (2H, app t), 7.17 (3H, app t), 2.67 (1H, m), 1.56 (2H, m), 1.29-1.22 (16H, m), 1.15 (1H, m), 0.87 (3H, t, 6.0Hz); ^{13}C-NMR (150MHz, $CDCl_3$) δ (ppm); 148.0 (s), 128.2 (d), 128.2 (d), 127.0 (d), 127.0 (d), 125.7 (d), 39.9 (d), 38.4 (t), 31.9 (t), 29.7 (t), 29.6 (t), 29.6 (t), 29.3 (t), 27.7 (t), 22.7 (t), 22.3 (q), 14.1 (q).

(R)-2-PhC3OH, + 13.1 (c 1.09, $CHCl_3$)

(S)-2-PhC3OH, - 13.9 (c 1.22, $CHCl_3$)

(R)-2-PhC3Br, + 17.5 (c 0.95, EtOH)

(S)-2-PhC3Br, - 17.8 (c 0.78, EtOH)

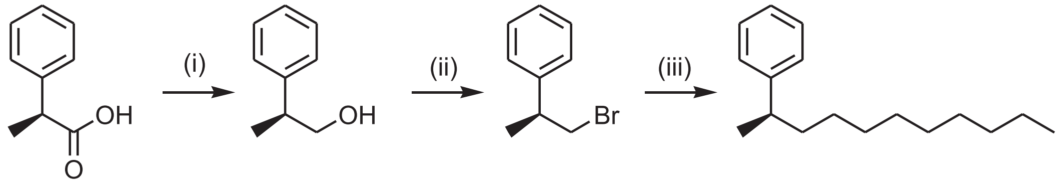

1b **2b** **3b** **4b**

Figure S6. Preparation of (*R*)-(−)-2-phenylundecane. Reagents: (i) $LiAlH_4$, THF; (ii) CBr_4, PPh_3, THF; (iii) C_8MgBr, NMP, Li_2CuCl_4, THF.

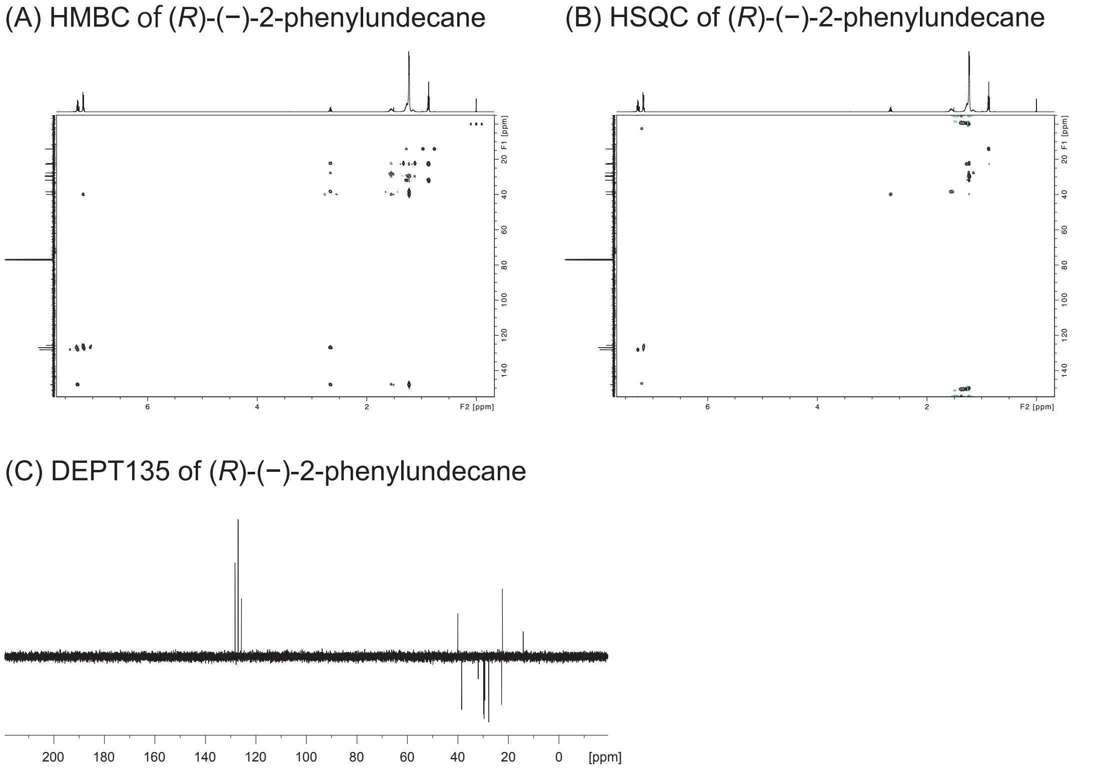

Figure S7. Results of chemical analyses for synthesized (*S*)-(+)-2-phenylundecane. (A) HMBC. (B) HSQC. (C) DEPT135.

Chiral separation of enantiomers of 2-phenylundecane

To identify mixture ratio of enantiomers of 2-phenylundecane in hexane fractions obtained from worker crude extracts, purification of this compound was performed by the following procedure. The hexane fractions derived from colonies A and B (each 100 μL) were applied to a silica gel column (30 mg, Wako gel C-200) and then collected every 100-μL hexane according to elution order (fractions 1 to 10). The 100-μL aliquots of each fraction were concentrated to 10 μL aliquots by using a gentle steam of nitrogen, and then subjected to gas chromatography (GC) analyses for confirming which fractions contained the target compound. GC analysis was performed on GC-2014 (Shimadzu) with a flame ionization detector (320°C). The injection port temperature was 300°C. The column used was a DB-1HT (15 m × 250 mm × 0.1 μm, Agilent Technologies). The carrier gas was helium, with a flow rate of 1.3 mL/min. Fractions (each 1 μL) were analysed in splitless mode with the temperature programmed to change from 50°C (5 min) to 320°C (5 min) at a rate of 20°C/min. 2-phenylundecane was identified by comparing the retention time with that of the synthesized racemic standard. 2-phenylundecane was contained in the fractions 2-4 (derived from colony A), or the fractions 3-5 (derived from colony B). Therefore, the fractions of each colony were mixed, and then each mixture (30 μL) was concentrated to 3 μL by using a gentle steam of nitrogen for the subsequent enantioselective analysis.

A chiral capillary column (Agilent CP-Chirasil Dex CB, 25 m x 0.25 mm, 0.25 μm) on an HP6890 gas chromatograph was used to separate enantiomers of 2-phenylpropan-1-ol and 2-phenylundecane. Helium was used as the carrier gas at 1 mL/min in the constant flow mode. For synthetic compounds, samples were injected at 230°C in the split mode (1:100) and detected by FID at 250°C. Oven temperature was 120°C (isothermal). For analyses of 2-phenylundecane from termite extracts, injection was made in the splitless mode (sampling time: 0.75 min) and oven temperature program was 50°C (1 min) - 120°C, 10°C/min and held for 102 min at 120°C. Chromatograms were analyzed by ChemStation ver. A10.01 (Agilent Technologies).

Retention times and enantiomeric excess for synthetic (*R*)- and (*S*)-2-phenylpropan-1-ol were 16.313 min (> 99% ee) and 16.683 min (> 99% ee), respectively. Those for synthetic (*R*)- and (*S*)-2-phenylundecane were 95.393 min (> 99% ee) and 94.499 min (> 99% ee), respectively. Under the oven temperature program for natural products, synthetic racemic 2-phenylundecane gave two peaks at 100.690 min (*S*-isomer) and 101.616 min (*R*-isomer). Two lots (colonies A and B) of the natural 2-phenylundecane from termites were analysed [colony A: 100.577 min (*S*-isomer) and 101.514 min (*R*-isomer), colony B: 100.618 min (*S*-isomer) and 101.655 min (*R*-isomer)], and both gave two isomers in *S*/*R* ratios of 1.88:1.00 and 1.62:1.00, respectively.

Text S3. Identification process of aggregation pheromone

We conducted the following bioassays to determine the active components of the aggregation pheromone used in the foraging workers of *R. speratus*.

Determining non-polar components

We first determined the compounds carrying the main activity in hexane and 10%DEE fractions. GC-MS analysis revealed that the hexane fractions contained short-chain *n*-alkanes (*n*-dodecane, *n*-tridecane, *n*-tetradecane, and *n*-hexadecane), 2-phenylundecane (2PhC11), and long-chain alkanes, and that the 10%DEE fractions also contained the long-chain alkanes, except for solvent-derived or contaminated compounds. Thus, the candidates of non-polar pheromone components were 2PhC11, and short- and long-chain alkanes. Our GC-MS analysis revealed that three straight *n*-alkanes [*n*-tricosane (C23), *n*-pentacosane (C25), and *n*-heptacosane (C27)] were available and the top three CHC components with the largest proportion of peak areas to total peak area in the chromatograms of hexane and 10%DEE fractions (Fig. 2 and Table S1). Therefore, we focused on C23, C25, and C27 among long-chain alkanes.

In the first step, we compared the activity of the blend of short-chain alkanes (SC), 2PhC11, and long-chain alkanes (LC). The mixed solutions were prepared with the components specific to the hexane fraction, each diluted in 1-mL hexane, in ratios that approximated the relative abundances in the hexane fractions. The SC solution was consisted of 60 ng of C12 (FUJIFILM Wako Pure Chemical Corporation), 40 ng of C13 (Nacalai tesque, Inc., Kyoto, Japan), 80 ng of C14 (Nacalai tesque, Inc.), and 40 ng of C16 (Nacalai tesque, Inc.)]. The 2PhC11 solution was consisted of 60 ng of (±)-2PhC11. The

LC solution was consisted of 580 ng of C23 (Sigma-Aldrich), 1340 ng of C25 (Tokyo Chemical Industry Co., Ltd.), and 860 ng of C27 (Honeywell Fluka). After that, a subtractive scheme was used to compare attractant activity of the mixed solution with three treatments that each lacked a different compound group, as well as control treatments. For each replication, a 10-μL mixed solution was added to the sample area, and the same amount of hexane was added to the control area. A hexane was dropped on the sample paper in the negative control treatment. The hexane fraction derived from the colony A was used as the positive control. We made 10 replications per colony for each treatment, using the workers from colonies C and F.

As a result, the workers evaded the mixture of all the three chemical groups (SC + 2PhC11 + LC treatment), but they were more attracted to the papers impregnated with the mixtures of the two groups (2PhC11 + LC, SC + LC, and SC + 2PhC11 treatments) than solvent papers, as well as the hexane fraction (Binomial test with Bonferroni correction, $P < 0.05$; Fig. S8). Nevertheless, there were no differences in mean of proportion of workers on sample papers among all treatments (a generalised linear mixed model [GLMM] followed by Tukey HSD test, $P > 0.05$) because the attraction levels of these sample solutions were low. These results mean that one (or two) of the three chemical groups contained substances that interfere the attraction activity of other compounds. Because LC was the main component of the 10%DEE fraction, which also had an aggregation activity, it was estimated that LC is included in the aggregation pheromone components. Comparing the mean proportion of workers aggregated at sample papers between the 2PhC11 + LC and SC + LC treatments, the former treatment tended to show higher aggregation level than the latter treatment. Therefore, 2PhC11 and LC were predicted to be the candidate components in hexane fraction.

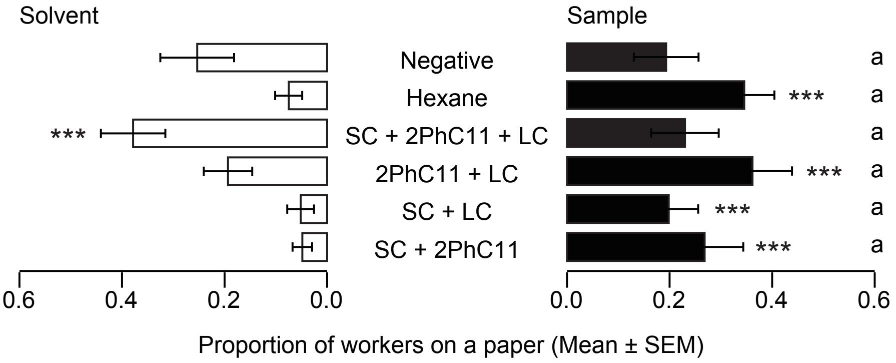

Figure S8. Attraction activities of candidate compounds in hexane fraction after 5 min. For details of bar plots, see Fig. 1. Abbreviations refer to main text. Asterisks mean significant differences between the total number of the workers on the sample papers and that of the workers on the solvent papers (binomial test with Bonferroni correction, no asterisk, no significance, ***: $P < 0.05$). Different alphabets indicate significant differences in the mean of proportion of workers on sample papers among treatments (GLMM followed by Tukey HSD test, $P < 0.05$). All combinations of candidate compounds except the mixture of SC, 2PhC11 and LC show significant attraction activities as well as the hexane fraction. Only the proportion of workers on the sample paper of the "2PhC11 + LC" treatment shows the same level as that of the hexane fraction.

Next, we confirmed whether 2PhC11 solely functions as the aggregation pheromone. The attractant activities of racemic 2PhC11 solution [1 µg of (±)-2PhC11 in 1-mL hexane] and its 10 to 100 times diluted solutions were tested. In addition, because the termite extracts contained both enantiomers of this compound [(+)-2PhC11: (−)-2PhC11 = 2:1], the activities of 2:1 and 1:2 mixtures of (+)-2PhC11 and (−)-2PhC11 (see Text S2) solutions [2:1 mixture: 200 ng of (+)-2PhC11 and 100 ng of (−)-2PhC11 in 1-mL hexane; 1:2 mixture: 100 ng of (+)-2PhC11 and 200 ng of (−)-2PhC11 in 1-mL hexane] were also tested at the same time. The workers derived from two colonies (colony I and J) were used. For each replication in all treatments except positive control, a 10-µl mixed solution was

added to the sample paper, and the same amount of hexane was added to the solvent paper. In the positive control, the 5-μl crude extract derived from colony I (1 worker equivalent) was added to the sample paper. In the negative control treatment, a hexane was dropped on the both papers. We used the workers from colonies I and J.

As a result, comparing total number of workers between sample and solvent papers, racemic 2PhC11 attracts workers at 0.1 and 10 ng as well as the crude extract within 5 min (binomial test with Bonferroni correction, $P < 0.001$; Fig. S9), although the proportion of workers on sample papers did not significantly differ among all treatments (GLMM followed by Tukey HSD test, $P > 0.05$). Therefore, it was estimated that 2PhC11 is able to attract workers by itself, even without volatile short-chain alkanes.

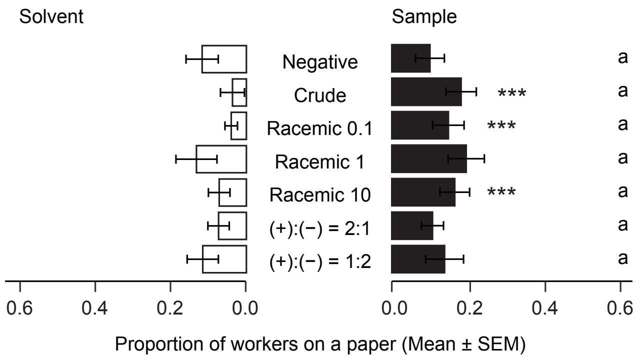

Figure S9. Aggregation activities of the mixtures of enantiomers of 2PhC11 after 5 min. For details of bar plots, see Fig. 1. Abbreviations refer to main text. Each treatment has 20 replications (10 replications × 2 colonies). Asterisks mean significant differences between the total number of the workers on the sample papers and that of the workers on the solvent papers (binomial test with Bonferroni correction, no asterisk: no significance, ***: $P < 0.001$). Different alphabets indicate significant differences in the mean of proportion of workers on sample papers among treatments (GLMM followed by Tukey HSD test, $P < 0.05$). (+):(−) means the mixture ratio of (+)- and (−)-2PhC11. Racemic 2PhC11 [equal to (±)-2PhC11] treatments (0.1 and 10 ng) attract workers as well as the crude treatment.

Furthermore, we hypothesized the long-chain alkanes may synergistically enhance the attractant activity with 2PhC11, because the long-chain hydrocarbons on termite cuticular surfaces, that is, cuticular hydrocarbons (CHCs) are necessary for nestmate recognition in termites [3]. To determine which long-chain alkanes carry the main activity in combination with 2PhC11, a subtractive scheme was used to compare activity of four-compounds' blend [60 ng of (±)-2PhC11, 580 ng of C23, 1340 ng of C25, and 860 ng of C27 in 1-mL hexane] with three treatments that each lacked a different alkane. C23, C25, and C27 were purchased from Sigma-Aldrich, Tokyo Chemical Industry Co., Ltd., and Honeywell Fluka, respectively. For each replication in all treatments except positive control, a 10-μl mixed solution was added to the sample paper, and the same amount of hexane was added to the solvent paper. The hexane fraction derived from the colony A was used as the positive control, and a 20-μl hexane fraction (1 worker equivalent) was added to the sample paper. A hexane was dropped on the sample paper in the negative control treatment. We used the workers from colonies C and F.

As a result, the 2PhC11 + C25 + C27 treatment showed attractant activity more than solvent papers, as well as the hexane fraction (binomial test with Bonferroni correction, $P < 0.001$; Fig. S10), but other hydrocarbon mixtures did not. However, the attraction levels of hexane fraction and the 2PhC11 + C25 + C27 treatment were still weak because there were no differences in the proportion of workers on sample papers among all treatments (GLMM followed by Tukey HSD test, $P > 0.05$). These results suggested that at least the activity of hexane fraction can be explained by three compounds (2PhC11, C25 and C27), which 2PhC11 attracts workers into the odour source and then C25 and C27 arrest them there.

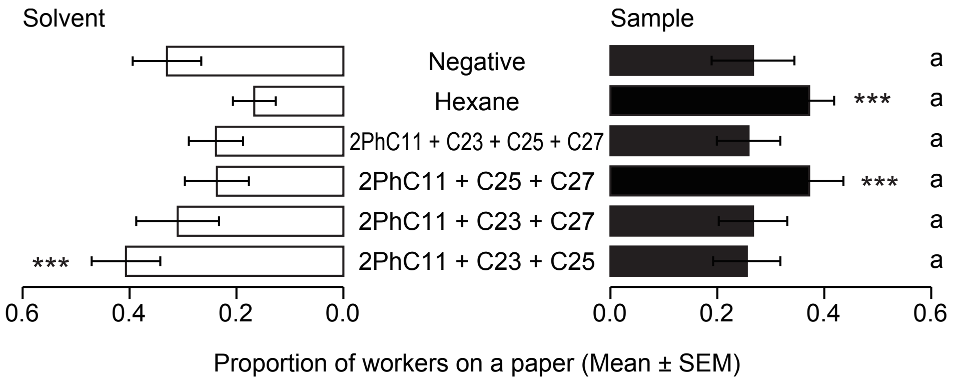

Figure S10. Attraction activities of the combinations of 2PhC11, C23, C25 and C27. For details of bar plots, see Fig. 1. Abbreviations refer to main text. Each treatment has 20 replications (10 replications × 2 colonies). Asterisks mean significant differences between the total number of the workers on the sample papers and that of the workers on the solvent papers (binomial test with Bonferroni correction, no asterisk: no significance, ***: $P < 0.001$). Different alphabets indicate significant differences in the mean of proportion of workers on sample papers among treatments (GLMM followed by Tukey HSD test, $P < 0.05$). Only the 2PhC11 + C25 + C27 treatment attracts workers as well as the hexane fraction.

Determining polar components

GC-MS analysis revealed that the 50%DEE fractions specifically contained myristic acid (MA), *trans*-vaccenic acid (tVA), and cholesterol (Ch), and that the acidic fractions specifically contained palmitic acid (PA) and oleic acid (OA). To narrow down candidate compounds in the 50%DEE fraction, a subtractive scheme was used to compare activity of blend [20 ng of MA (FUJIFILM Wako Pure Chemical Corporation), 400 ng of tVA, and 1160 ng of Ch (FUJIFILM Wako Pure Chemical Corporation) in 1-mL hexane] with three treatments that each lacked a different component, as well as negative and positive control treatments. We used the workers from 2 termite colonies (C and E). The ratio of candidate compounds was approximated the relative abundances in 50%DEE fraction. For each replication in all treatments except positive control, a 10-µl mixed

solution was added to the sample paper, and the same amount of hexane was added to the solvent paper. The 50%DEE fraction derived from the colony A (20 µl, 1 worker equivalent) was added to the sample paper as the positive control. A mixture of hexane and DEE (= 1:1) was dropped on the both papers in the negative control treatment.

Bioassay result showed that all mixture except for the MA + tVA + Ch treatment attracted workers as well as the 50%DEE fraction (binomial test with Bonferroni correction, $P < 0.05$; Fig. S11) in comparison with solvent papers. Although the tVA + Ch and MA + tVA treatments showed higher mean proportion of workers on sample papers than other treatments (GLMM followed by Tukey HSD test, $P < 0.05$), but the proportion in the tVA + Ch treatment tended to show the highest value among all treatments. This suggested that tVA and Ch carry the main activity in 50%DEE fraction. Since both tVA and Ch are not volatile, it is estimated that tVA and Ch arrested workers passing by.

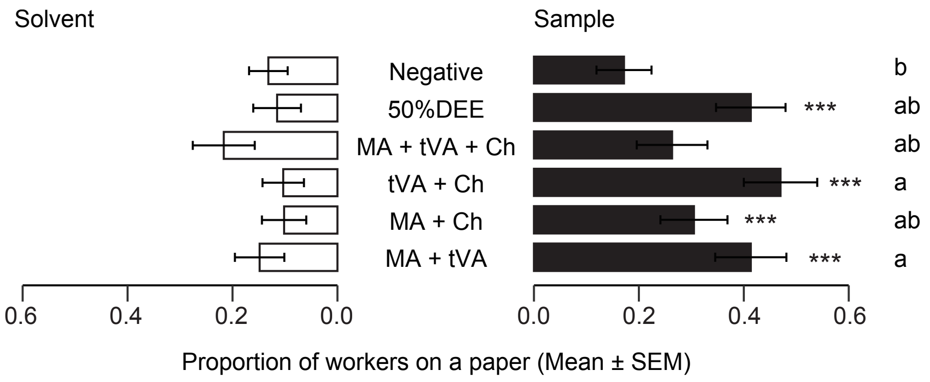

Figure S11. Attraction activities of candidate compounds in the 50% DEE fraction. For details of bar plots, see Fig. 1. Abbreviations refer to main text. Each treatment has 20 replications (10 replications × 2 colonies). Asterisks mean significant differences between the total number of the workers on the sample papers and that of the workers on the solvent papers (binomial test with Bonferroni correction, no asterisk: no significance, ***: $P < 0.001$). Different alphabets indicate significant differences in the mean

of proportion of workers on sample papers among treatments (GLMM followed by Tukey HSD test, $P < 0.05$). All mixtures of candidate compounds except the "MA + tVA + Ch" show significant attractant activities as well as the 50% DEE fraction after 5 min, but in terms of the proportion of workers on the sample paper, the "tVA + Ch" treatment exceeds the 50% DEE fraction.

Next, to determine candidate compounds in the acid fraction, we prepared the following four mixed solutions: (±)-2PhC11: C25: C27: tVA: Ch = 0.6:13.4:8.6:4:11.6, 0.6:13.4:8.6:4:11.6:200, 0.6:13.4:8.6:4:11.6:21.9, and 0.6:13.4:8.6:4:11.6:200:21.9 (unit: ng per 10-µL hexane). PA was purchased from Nacalai tesque Inc., and OA was purchased from Tokyo Chemical Industry Co., Ltd. Using the workers from 2 colonies (E and G), we compared the attractant activities among the above four mixed solutions, the negative control (hexane), and the positive control (crude extract). The ratio of palmitic and oleic acids was approximated the relative abundances in acid fractions. For each replication in all treatments except positive control, a 10-µl mixed solution was added to the sample paper, and the same amount of hexane was added to the solvent paper. In the positive control, the 5-µl crude extract derived from colony A (1 worker equivalent) was added to the sample paper.

As a result, the 2PhC11 + C25 + C27 + tVA + Ch + PA treatment showed a significant attractant activity in comparison with solvent papers (binomial test with Bonferroni correction, $P < 0.05$, Fig. S12), but other mixtures of candidates did not attract workers. Also, the 2PhC11 + C25 + C27 + tVA + Ch + PA treatment showed higher mean proportion of workers on sample papers than other mixtures (GLMM followed by Tukey HSD test, $P < 0.05$). Therefore, it was estimated that the minimum components of aggregation pheromone in *R. speratus* comprise the mixture of 2PhC11, C25, C27, tVA, PA

and Ch. However, the mixture ratio needed to be improved to increase the attraction activity, because the above mixture ratio of these six compounds [(±)-2PhC11: C25: C27: tVA: PA: Ch = 0.6:13.4:8.6:4:200:11.6] did not show attractant activity of the same level as the worker crude extract.

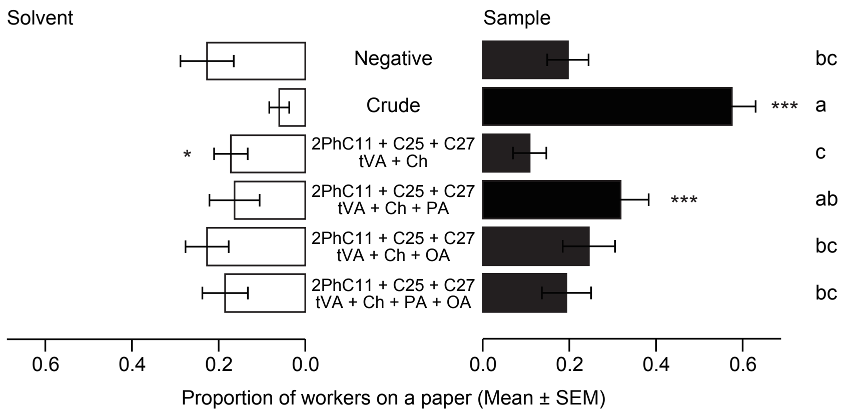

Figure S12. Attractant activities of the mixture of (±)-2PhC11, C25, C27, tVA, and Ch in combination with PA and/or OA. For details of bar plots, see Fig. 1. Abbreviations refer to main text. Each treatment has 20 replications (10 replications × 2 colonies). Asterisks mean significant differences between the total number of the workers on the sample papers and that of the workers on the solvent papers (binomial test with Bonferroni correction, no asterisk: no significance, *: $P < 0.05$, ***: $P < 0.001$). Different alphabets indicate significant differences in the mean of proportion of workers on sample papers among treatments (GLMM followed by Tukey HSD test, $P < 0.05$). Only the "2PhC11 + C25 + C27 + tVA + Ch + PA" treatment shows a significant attractant activity as well as the crude treatment after 5 min.

Mixture ratio and attractant activity

To find a better mixture ratio for attracting more workers, we tested the attraction activity of different mixture ratios of the pheromone components. We compared an activity of the normative blend [(±)-2PhC11: C25: C27: tVA: PA: Ch = 0.6:13.4:8.6:4:200:11.6 (unit: ng per 10-μL hexane)] with six treatments that the quantity of any one of components

was 10 times greater than the normative blend, as well as negative (hexane) and positive control treatments. In this assay, we used the workers from two colonies (E and G). For each replication in all treatments except positive control, a 10-μl mixed solution was added to the sample paper, and the same amount of hexane was added to the solvent paper. In the positive control, the 5-μl crude extract derived from colony A (1 worker equivalent) was added to the sample paper.

As a result, comparing the proportion of workers on sample papers among treatments, only the 10-fold 2PhC11 treatment showed the lowest worker attractant activity (GLMM followed by Tukey HSD test, $P < 0.05$). However, comparing total number of workers between sample and solvent papers in each treatment, the workers were significantly attracted not only by the crude extract and the normative blend but also by the 10-fold C27 treatment and the 10-fold tVA treatment (binomial test with Bonferroni correction, $P < 0.05$, Fig. S13). In particular, the treatment containing 10-fold tVA tended to attract more workers into the sample paper than other treatments except for the crude extract. Therefore, it was estimated that the mixture ratio of the 10-fold tVA treatment (2PhC11: C25: C27: tVA: PA: Ch = 0.6:13.4:8.6:40:200:11.6) was better than other mixture ratios.

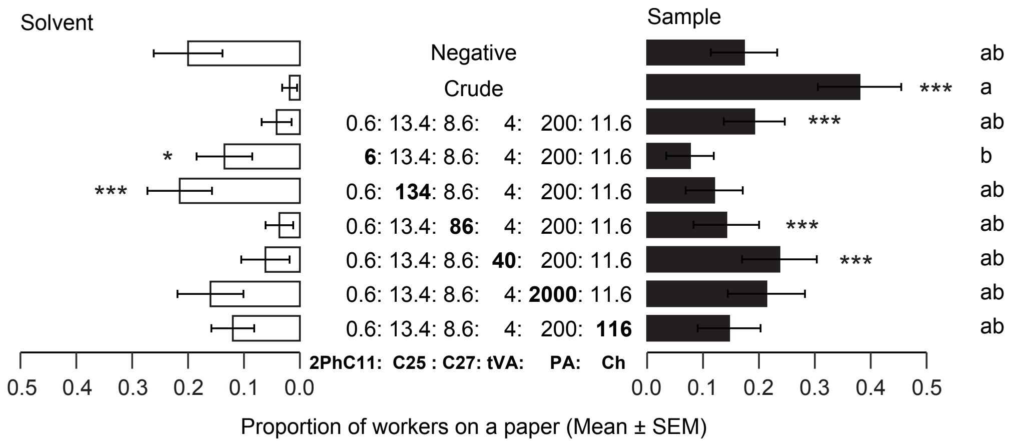

Figure S13. Assay results of mixtures of (±)-2PhC11, C25, C27, tVA, PA and Ch with different mixing ratios after 5 min. For details of bar plots, see Fig. 1. Each treatment has 20 replications (10 replications × 2 colonies). Asterisks mean significant differences between the total number of the workers on the sample papers and that of the workers on the solvent papers (binomial test with Bonferroni correction, no asterisk: no significance, *$P < 0.05$, ***: $P < 0.001$). Different alphabets indicate significant differences in the mean of proportion of workers on sample papers among treatments (GLMM followed by Tukey HSD test, $P < 0.05$). Mixture ratios displayed between bar plots indicate the ratios of the above six compounds [unit: ng/paper]. The normative mixture (0.6:13.4:8.6:4:200:11.6) and the treatments containing 10-fold dose of C27 or tVA show significant attractant activities. In particular, the mean proportion of workers on sample papers in the treatment containing 10-fold dose of tVA was slightly higher than other treatments except for the crude extract.

Also, we compared the pheromone activities among the blends that (+)- and (−)-2PhC11 were mixed in a ratio of 2:1, 1:1, or 1:2 with other five pheromone components, because the worker crude extracts contained both (+)- and (−)-2PhC11 in a ratio of 2:1 (see Result section). We prepared the following three mixed solutions: (+)-2PhC11: (−)-2PhC11: C25: C27: tVA: PA: Ch = 0.3:0.3:13.4:8.6:40:200:11.6 (racemic-based mix), 0.2:0.4:13.4:8.6:40:200:11.6 [(−)-2PhC11-biased mix], and 0.4:0.2:13.4:8.6:40:200:11.6 [(+)-2PhC11-biased mix] (unit: ng per 10-µL hexane). We compared the attractant activities

among the above mixed solutions, the negative control (hexane), and the positive control (crude extract from colony A). In this assay, we used the workers from two colonies (E and H). For each replication in all treatments except positive control, a 10-µl mixed solution was added to the sample paper, and the same amount of hexane was added to the solvent paper. In the positive control, the 5-µL crude extract (1 worker equivalent) was added to the sample paper. In the negative control treatment, a hexane was dropped on the both papers.

As a result, comparing total number of workers between sample and solvent papers, the racemic-based mix [equal to a 0.6:13.4:8.6:40:200:11.6 mixture of (±)-2PhC11, C25, C27, tVA, PA and Ch] and the (+)-2PhC11-biased mix showed significant attractant activity as well as the crude extract (binomial test with Bonferroni correction, $P < 0.05$, Fig. S14). Furthermore, comparison of mean proportion of workers on sample papers among treatments also showed the same trend (GLMM followed by Tukey HSD test, $P < 0.05$). Notably, the proportion of workers on sample papers of the former mixture showed the highest attractant activity among all treatment.

Consequently, we estimated that the effective mixture ratio of the artificial aggregation pheromone is (±)-2PhC11: C25: C27: tVA: PA: Ch = 0.6:13.4:8.6:40:200:11.6.

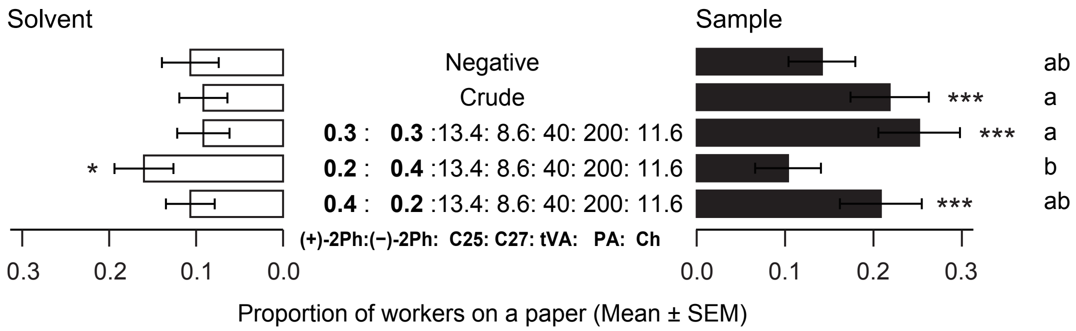

Figure S14. Assay results of mixtures of (+)-2PhC11, (−)-2PhC11, C25, C27, tVA, PA and Ch, which changed only the mixing ratio of (+)-2PhC11 and (−)-2PhC11 after 5 min. For details of bar plots, see Fig. 1. Each treatment has 20 replications (10 replications × 2 colonies). Asterisks mean significant differences between the total number of the workers on the sample papers and that of the workers on the solvent papers (binomial test with Bonferroni correction, no asterisk: no significance, *$P < 0.05$, ***: $P < 0.001$). Different alphabets indicate significant differences in the mean of proportion of workers on sample papers among treatments (GLMM followed by Tukey HSD test, $P < 0.05$). “2Ph” is the abbreviation of 2PhC11. The racemic-based mix (0.3:0.3:13.4:8.6:40:200:11.6) and the (+)-2PhC11-biased mix (0.4:0.2:13.4:8.6:40:200:11.6) showed significant attractant activity as well as the crude extract, although the proportion of workers on sample papers of the former mixture exceeded that of the crude extract.

Supplementary references

1. Abate, A. *et al.* Chirality and fragrance chemistry: stereoisomers of the commercial chiral odorants *Muguesia* and *Pamplefleur*. *J. Org. Chem.* **70**: 1281-1290 (2005).
2. Prins, L. J., Hulst, R., Timmerman, P. & Reinhoudt, D. N. Diastereoselective noncovalent synthesis of hydrogen-bonded double-rosette assemblies. *Chem-Eur. J.* **8**: 2288-2301 (2002).
3. Takematsu, Y. & Yamaoka, R. Cuticular hydrocarbons of *Reticulitermes* (Isoptera : Rhinotermitidae) in Japan and neighboring countries as chemotaxonomic characters. *Appl. Entomol. and Zool.* **34**:179–188 (1999).
4. Yadav J.S. & Mysorekar S. V. A facile conversion of tertiary alcohols to olefins. *Synthetic Commun.* **19**: 1057-1060 (1989).
